# Mettl14-mediated m6A modification enhances the function of Foxp3^+^ regulatory T cells and promotes allograft acceptance

**DOI:** 10.3389/fimmu.2022.1022015

**Published:** 2022-10-19

**Authors:** Yanzhuo Liu, Yinglin Yuan, Zili Zhou, Yuanyuan Cui, Yan Teng, Hao Huang, Hao Yuan, Yanling Zhang, Lu Yang, Gaoping Zhao

**Affiliations:** ^1^ Department of Gastrointestinal Surgery, Sichuan Academy of Medical Sciences & Sichuan Provincial People’s Hospital, School of Medicine, University of Electronic Science and Technology of China, Chengdu, China; ^2^ Clinical Immunology Translational Medicine Key Laboratory of Sichuan Province, Sichuan Provincial People’s Hospital, University of Electronic Science and Technology of China, Chengdu, China; ^3^ Institute of Neurology, Sichuan Provincial People’s Hospital, School of Medicine, University of Electronic Science and Technology of China, Chengdu, China

**Keywords:** N6-methyladenosine, Mettl14, Treg function, transplantation, allograft acceptance

## Abstract

N6-methyladenosine (m6A), the most prevalent form of internal mRNA modification, is extensively involved in Treg cells differentiation and function. However, the involvement of m6A in functional Treg cells for transplantation tolerance remains to be elucidated. By using an experimental transplantation mouse model, we found that m6A levels in Treg cells were altered during the induction of transplant tolerance by performing a dot blotting assay. Subsequently, we used the heterogenic Treg-specific Mettl14 knockout mice (Foxp3-Mettl14^f/+^ cKO) to reduce METTL14 expression and performed islets allograft transplantation. Our result revealed that reduced expression of METTL14 prevented Treg cells expansion and promoted the infiltration of CD4^+^ and CD8^+^ T cells around the allograft, which led to rapid allograft rejection in Foxp3-Mettl14*^f/+^* cKO mice. The expression of regulatory cytokines including IL-10 and TGF-β was significantly decreased in Foxp3-Mettl14*^f/+^* cKO mice, and the suppressive function of Treg cells was also abrogated. In addition, an analysis of RNA-seq data revealed that the SOCS family (SOCS1, SOCS2 and SOCS3) is the subsequent signaling pathway affected by the METTL14 mediated m6A modification in Treg cells to modulate the suppressive function after transplantation. Taken together, our study showed for the first time that the METTL14-mediated m6A modification is essential for the suppressive function of Treg cells in transplantation and may serve as a regulatory element of Treg cell-based therapy in transplant medicine.

## Introduction

Organ/tissue transplantation is one of the most effective therapeutic options for end-stage organ failure. Successful allograft transplantation is constantly plagued by the challenge of acute and chronic cell-mediated allograft rejection. This process is generally mediated by indirect or direct forms of antigen recognition and activated T-cell response (1–3). T cells respond to foreign (allogeneic) MHC molecules in the same way as to any foreign proteins, which include cytokine secretion, cell proliferation as well as differentiation. This process leads to the accumulation of a large number of effector T cells and macrophages, which are the main mediators in the process of graft rejection (4). Regulatory T cells (Treg cells) are essential for the maintenance of immune homeostasis. The main feature of Treg cells is the ability to regulate or suppress the proliferation and function of other effector T cells (5). Mechanistically, cell death or anergy of other effector T cells can be induced in the presence of Treg cells (4). Thus, many efforts have been made to preserve the number and function of Treg cells and achieve long-term graft survival after transplantation with an unsatisfactory outcome.

Transcriptional regulation is coordinated with epigenetic modifications, which affect gene expression and function (6). Previous studies have shown that RNA modification, particularly N6-methyladenosine (m6A), is the most prevalent reversible methylation modification of eukaryotic mRNA (7, 8). The main m6A regulators include methyltransferase ‘writers’, demethylases ‘erasers’, and the m6A-binding protein ‘readers’ of the RNA chemical mark (9). Based on accumulating evidence, the abundance of the m6A modification in immune cells correlates with multiple immune responses (10). In this regard, the m6A ‘writer’ enzyme METTL3 plays an essential role in dendritic cells and T cells during cellular development, activation, and maturation, as well as influencing Treg cells function and stability (11). These previous studies evaluated the significance of the m6A modification in the progression of cell-mediated immunity. Another study also indicated that METTL14 deficiency in T cells induces spontaneous colitis in mice due to dysfunctional Treg cells (12). Since functional Treg cells are closely related to controlling the development of rejection or tolerance after transplantation, it is necessary to explore the potential roles of the m6A modification in Treg cells after transplantation.

Our group developed multiple allograft transplantation models, including islet, heart, skin, and kidney models, and these models have been used to explore the mechanisms of immune tolerance, including the suppressive function of Treg cells (13, 14). Based on our previous work in transplantation, we generated mice carrying a conditional deletion of the Mettl14 allele in mice expressing Cre recombinase under the control of the Foxp3 promoter, to delete or knockdown Mettl14 in Treg cells. We used Foxp3-Mettl14^f/+^ cKO mice to evaluate the role of m6A modification in functional Treg cells after transplantation.

## Materials and methods

### Mice and genotyping

Mettl14^f/f^ mice with a C57BL/6J background were generated by changing the sequences flanking the Mettl14 gene using the CRISPR/CAS9-based genome-editing system. Then, these mice were crossbred with Foxp3-Cre mice (Jackson Labs) to generate Treg-specific heterozygote (Foxp3-Mettl14^f/+^), homozygote (Foxp3-Mettl14^f/f^) and littermate control mice. The PCR products obtained, using 5’loxp-F and 5’loxp-R primers were separated using agar gel electrophoresis. A 452-bp band was detected as Cre-Foxp3. A 250-bp band was detected the loxp sequence. Whereas a 198-bp band was observed in the wild-type sample, which identified littermate controls mice (Mettl14^f/f^).

BALB/c mice were used as pancreatic tissue donors. Wild type mice (C57BL/6J background), Foxp3-Mettl14^f/+^ cKO mice and littermate controls were used as recipients. The animal procedures were compliant with the Institutional Animal Care and Use Committee of the University of Electronic Science and Technology of China.

### Islet transplantation

Diabetes was induced in wild-type (C57BL/6J background), littermate controls and Foxp3-Mettl14^f/+^ cKO mice by administering a single intraperitoneal injection of streptozotocin (200 mg/kg, Sigma Aldrich, St. Louis, MO, USA). The established mouse model of type 1 diabetes was confirmed as a transplant recipient by monitoring the blood glucose level [> 18.8 mmol/L (400 mg/dL)] for at least 2 consecutive days. Pancreatic islets were separated from the pancreata of donor (BALB/c) mice at a ratio of three pancreata per recipient. Briefly, islets derived from BALB/c mice were isolated by using 1.5 mg/ml cold collagenase for digestion (Roche, Mannheim, Germany) and then purified by Ficoll discontinuous gradients centrifugation (densities: 1.11, 1.096, 1.066). The cells at the interface between the 1.096 and 1.066 layers were the separated islet cells. Then, the islets were transplanted into the capsule of kidney as described previously (15). Graft rejection was defined as non-fasting blood glucose levels > 11.1 mmol/l for two consecutive days after a period of euglycemia. Induced transplant tolerance (long-term allograft survival occurred) was defined as the non-fasting blood glucose levels less than 11.1 mmol/L (200 mg/dL) for more than 90 days. Nephrectomy was performed at day 90 post-islet transplantation to determine whether the euglycemia was graft dependent.

### Isolation of Treg cells and CD4^+^ naïve T cell

Mononuclear suspensions were recovered from mouse spleens and passed the cells through a 70 μm nylon mesh. Erythrocytes were lysed with ammonium chloride buffer and counted using a hemocytometer. Mouse Treg cells was isolated using a CD4^+^CD25^+^ Regulatory T cell Isolation Kit according to the manufacturer’s protocols (Miltenyi, Bergisch Gladbach, Germany) (16, 17). Briefly, cells were isolated using a two-step procedure based on depleting samples of non-CD4^+^ T cells followed by positive selection of Treg cells.

Moreover, we purified mouse spleen CD4^+^ naïve T cell using a CD4^+^ Naïve T cell Isolation Kit (#130-104-453, Miltenyi, Bergisch Gladbach, Germany) (18).

The purities of the sorted Treg cells or CD4^+^ naïve T-cell population were always 90% as confirmed by flow cytometry.

### Real-time quantitative PCR (RT-qPCR)

Treg cells were isolated as previously described. Total RNA was extracted from Treg cells using TRIzol (Invitrogen, Carlsbad, CA, USA). A reverse transcriptase reaction was performed using a HiScript III RT SuperMix kit (Vazyme, Shanghai, China). Subsequently, ChamQ Universal SYBR qPCR Master Mix (Vazyme, Shanghai, China) was used to perform the real-time quantitative PCR assay. Relative gene expression was quantified using the 2^-△△CT^ method. The Gapdh served as a reference gene. Each sample was assayed in triplicate. The following primer sets were used for the RT-qPCR (**Table 1**).

**Table 1:** The primer sets for RT-qPCR are shown below.

GAPDH	Forward,5’-CTACACTGAGGACCAGGTTGTC-3’
	Reverse,5’-GTT ATT ATG GGG GTCTGG GAT GG-3’
FOXP3	Forward, 5’- GGTACACCCAGGAAAGACAG -3’
	Reverse, 5’- ATCCAGGAGATGATCTGCTTG -3’
Mettl14	Forward, 5’-CTGAGAGTGCGGATAGCATTG-3’
	Reverse, 5’-GAGCAGATGTATCATAGGAAGCC-3’
Mettl3	Forward, 5’-CTGAGAGTGCGGATAGCATTG-3’
	Reverse, 5’- ATCCAGGAGATGATCTGCTTG -3’
ALKBH5	Forward, 5’-GCATACGGCCTCAGGACATTA-3’
	Reverse, 5’-TTCCAATCGCGGTGCATCTAA-3’
FTO	Forward, 5’-GACACTTGGCTTCCTTACCTG-3’
	Reverse, 5’-CTCACCACGTCCCGAAACAA-3’
SOCS1	Forward, 5’-CCTTTTCGAGCTGCTGGAG-3’
	Reverse, 5’-TACCGGGTTAAGAGGGATGC-3’
SOCS2	Forward, 5’-GCAAGGATAAACGGACAGGC-3’
	Reverse, 5’-GGTAAAGGCAGTCCCCAGAT-3’
SOCS3	Forward, 5’-ACCAAGAACCTACGCATCC-3’
	Reverse, 5’-GTGGCAAAGAAAAGGAGGGG-3’

### RNA m6A dot-blot assays

Total RNA was extracted from Treg cells as described above. The mRNA samples were dissolved in 3 volumes of RNA incubation buffer and denatured at 65°C for 5 min. The samples were divided into subgroups of 200 ng and were loaded onto an NT membrane (Millipore, Billerica, MA, USA). The membrane was UV cross-linked for 1200 w and washed with TBST. Then, it was stained with 0.02% methylene blue (Sangon Biotech, China), followed by scanning to indicate the total content of input RNA. After blocking with 5% non-fat milk, the membrane was incubated with a specific m6A antibody (1:5000, Millipore, Billerica, MA, USA) overnight at 4 °C. Dot blots were hatched with HRP-conjugated anti-mouse immunoglobulin G (IgG) for 1 hour before visualization by using an HRP chemiluminescence kit (EMD Millipore; Billerica, MA, USA).

### Western blot analysis

Treg cells were isolated as described above, total protein was extracted from cells using pre-cooled RIPA buffer (Solarbio, Beijing, China) containing protease and phosphatase inhibitors (Thermo Scientific, USA). Protein concentrations were quantified with a Bicinchoninic Acid Protein Assay Kit (Thermo Scientific, USA). Equal amounts of protein samples were separated by sodium dodecyl sulfate-polyacrylamide gel electrophoresis (SDS-PAGE; 10% or 15%) and then transferred to PVDF membranes (Millipore, Burlington, MA, USA). After blocking with 5% non-fat milk in TBST for 1 h, the membranes were incubated with the corresponding primary antibodies at 4 °C overnight. The membranes were washed with TBST three times, followed by an incubation with HRP-conjugated secondary antibodies for 1 hour at room temperature. The primary antibodies used in this study as follows. Foxp3 (1:2000, Abcam, Cambridge, MA, USA), METTL14 (1:2000, Peprotech, Rocky Hill, NJ,USA), METTL3 ((1:2000, Peprotech, Rocky Hill, NJ,USA) ALKBH5 ((1:2000, Peprotech, Rocky Hill, NJ,USA), FTO ((1:2000, Peprotech, Rocky Hill, NJ, USA), SOCS1-3 ((1:2000, HUABIO, Hangzhou) and β-Actin ((1:2000, Peprotech, Rocky Hill, NJ, USA).

### Immunotherapy

Recipient mice received 100 µg anti-mouse CD45RB antibody (Bio X Cell, West Lebanon, NH, USA) delivered by intraperitoneal injection on days 0, 1, 3, 5, and 7 after transplantation. In addition, recipient mice also receive 500 μg anti-mouse of an CD40L antibody (MR-1, Bio X Cell, West Lebanon, NH, USA) delivered by intraperitoneal injection on days 2, 4 and 7 following transplantation. The dose and duration were selected according to previously published studies (19, 20).

### Immunohistochemistry (IHC)

The engrafted kidneys were harvested from Foxp3-Mettl14*^f/+^* cKO mice and littermate controls after transplantation by nephrectomy. The engrafted kidney was preserved in 10% formalin overnight. Immunostaining procedures were performed using a standard approach (21). Briefly, 4 to 6 μm tissue sections were deparaffinized and washed three times with PBS. These sections were sealed with blocking buffer (Thermo Fisher Scientific, USA). Then, primary antibodies against CD4^+^ and CD8^+^ (1:200, Abcam, USA) were applied to these sections and incubated in a humidified chamber at room temperature for 2 hours. Next, these sections were incubated with Sav-HRP conjugates (Cell Signaling Technology, USA) at room temperature for 30 min in the dark. Afterward, these sections were incubated with biotinylated secondary antibodies (Cell Signaling Technology, USA) at room temperature for 1 hour. These sections were dehydrated with 95% ethanol and 100% ethanol. Next, a DAB substrate solution (Cell Signaling Technology, USA) was added to these sections to reveal the color of IHC staining. Finally, images of these sections were obtained under a microscope and analyzed with Image Pro Plus software. This assay was repeated at least three times.

### Cell stimulation and flow cytometry

Mononuclear suspensions were recovered from the spleen and renal draining lymph nodes (RDLNs) as previously described (22). One million cells were suspended in a buffer containing 0.1% azide and 2% fetal bovine serum. For intracellular cytokine staining, cells were stimulated with the Stimulation Cocktail Kit containing PMA, ionomycin and protein transport inhibitor (1:500, eBioscience, San Diego, CA, USA)) at 37 °C for 3-5 hours. The membrane antibodies were incubated on ice for 20 min, including CD3-APC-Cy7, CD4-BB700, and CD25-APC antibodies. For intracellular staining of Foxp3, IFN-γ, IL-4 and IL-17a, cells were fixed and permeabilized using fixation and permeabilization buffer (eBioscience, San Diego, CA, USA) and incubated with the intracellular fluorescence-labeled antibodies, including anti-IFN-γ-PE, anti-IL-4-PerCP-cy5.5, anti-IL-17a-PE and anti-Foxp3-PE. All antibodies were purchased from BD Biosciences (San Diego, CA, USA). All samples were run on a BD flow cytometer and analyzed using Flow Jo analysis software (Tree Star Inc.).

### Enzyme-Linked Immunosorbent Assay (ELISA)

The serum was harvested from recipients in different groups and stored at −80°C until analysis. The concentrations of cytokines including IL-10, TGF-β, IL-2 and IFN-γ in serum were measured using ELISA kit (Neobioscience, China, Shenzhen) according to the manufacturer’s protocol.

### Cell and short interfering RNA (siRNA) transfection

Treg cells (2×10^6^) were isolated as described above and plated into each well of a 24-well plate. The siRNA and negative control siRNA were transfected with as duplexes using the Lipofectamine 2000 transfection reagent (Invitrogen, Carlsbad, CA, USA) (Mettl14 siRNAs; sense: 5*’*-GGGAGAGUAUGCUUGCGAATT 3*’*-UUCGCAAGCAUACUCUCCCTT). After the cells were transfected for 6 hours at 37°C in 5% CO^2^ with Opti-MEM medium (Invitrogen, Carlsbad, CA, USA), the medium in each well was replaced with complete RPMI 1640 without antibiotics and incubated for 48 h at 37 ˚C before subsequent experiments. The protein expression of METTL14 was detected using western blotting.

### CFSE labeling and Treg cells suppression assays

As described previously, Treg cells were isolated from Foxp3-Mettl14*^f/+^* cKO mice and littermate controls after transplantation and were used as suppressor cells. The CD4^+^ naïve T cells from C57/6J mice were used as responder cells. For the assay, CD4^+^ naïve T cells (1×10^6^ cells/ml in PBMI) were freshly stained with 2 μM carboxyfluorescein diacetate succinimidyl ester (CFSE, Invitrogen, Carlsbad, CA, USA) for 15 min at room temperature and cultured in round-bottom 96-well plates containing anti-CD3 (3 μg/ml, BD Bioscience) and anti-CD28 (5 μg/ml, BD Bioscience) monoclonal antibodies (1–2×10^5^/well). For the suppression assay, Treg cells were co-cultured with labeled-CD4^+^ naïve T-cells at a certain ratio, namely 0:1, 1:1 1:2, and 1:4. After five days, the ratio of proliferation was analyzed by flow cytometry.

### Mixed lymphocyte reaction assay

Enriched Tregs cells were isolated as previously described. CD4^+^ naïve T-cells (0.3×10^6^) as responder was isolated from spleens and labeled with CFSE. BALB/c splenocytes (2×10^5^) were irradiated (6 Gry) as allogeneic stimulator cells. In general, the responder cells and stimulator cells were resuspended in Iscove modified Dulbecco’s medium (IMDM, HyClone, Logan, UT, USA) in 96-well round-bottom plates, and anti-CD3/CD28 antibody (eBioscience, T-activator CD3/CD28 Dynabeads, (0.5μg/ml) was added (23–25). Treg cells (0.2×10^4^) were subsequently added to each well. These cells were cultured at 37°C for 5 days, and proliferation was monitored using flow cytometry.

### RNA sequencing (RNA-seq)

As described above, Treg cells were isolated from Foxp3-Mettl14^f/+^ cKO mice and littermate controls on day 7 post-transplantation. Total RNA was extracted with TRIzol and mRNA was then separated by using a Dynabeads mRNA Purification Kit (Invitrogen). Standard Illumine HiSeq2000 sequencing was applied for sequencing. Raw RNA-sequencing reads were aligned to the mouse genome (mm10) with TopHat. Genes were considered significantly differentially expressed if the fold change was greater than 1.5-fold and less than 0.1. A GO enrichment analysis and KEGG pathways enrichment analysis were performed using online bioinformatics tools.

### Statistical analysis

In all the bar graphs, data are reported as the means ± SD, unless described. Statistical analyses were performed using GraphPad Prism 6 software. Two-tailed unpaired Student’s t-test was used to compare data between two groups. Graft survival was analyzed using the Kaplan–Meier method, and survival curves were compared using the log-rank test. P values ≤ 0.05 were considered significant (P*<0.05, **P< 0.01, ***P< 0.001 and ****P<0.0001); *P* values >0.05 were considered nonsignificant (n.s.). FlowJo software (Treestar) was used to analyze all the flow cytometry data.

## Results

### The level of the m6A modification in Treg cells correlates with allograft tolerance

**Figure 1:**
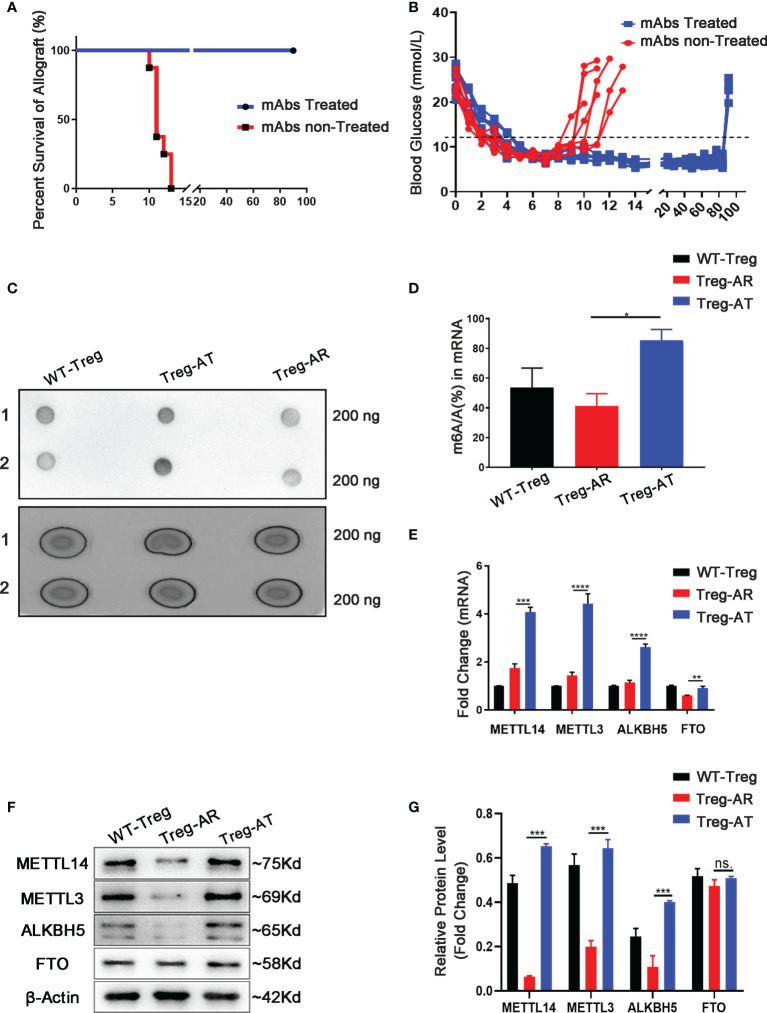
The level of the m6A modification increases in Treg cells from allograft tolerant group. **(A)** Islet allografts (BALB/c mice to C57BL/6J mice) displayed long-term graft survival in all recipients (n=7, 100%) under anti-CD45RB and anti-CD40L monoclonal antibodies (mAbs) treatment (blue line). Without mAbs treatment, the islet allografts were rejected within 13 ± 2.2 days (red line). **(B)** Blood glucose levels in recipients represent the function of islets allograft post-transplantation. Diabetic mice transplanted islets and treated with mAbs maintained the long-term allograft function as an allograft tolerance (AT) group (blue line), and splenic-derived Treg cells were isolated on day 90 days after transplantation as Treg-AT cells. Without mAbs treatment, the recipients rejected islets allograft rapidly and was referred to as the allograft rejection (AR) group (red line). The splenic-derived Treg cells were isolated from the recipients with two successive non-fasting blood glucose concentrations exceeding 11.1 mmol/L as Treg-AR cells. **(C, D)** The global m6A level in total RNA isolated from Treg cells (including Treg-AR cells and Treg-AT cells) was measured using dot blotting. **(E)** The mRNA levels of m6A regulators were measured by using qPCR, and METTL14, METTL3, and ALKBH5 levels were significantly decreased in the Treg-AR group, compared to those in the Treg-AT group. **(F, G)** Western blot analysis indicated that the METTL14, METTL3, and ALKBH5 protein levels were significantly decreased in the Treg-AR group compared to those in the Treg-AT group. The data are shown as the means ± SD and are representative of three separate experiments. The data in **(E)** depict the mean mRNA expression normalized to the Gapdh mRNA. The data in **(G)** depict the mean protein expression normalized to the gene β-Actin The statistical analysis was performed with an unpaired Student’s t-test (two-tailed). ****P<0.0001, ***P<0.001, **P<0.01, *P<0.05. *P* values >0.05 were considered nonsignificant (n.s.).

We isolated islets from BALB/c mice and transplanted them under the kidney capsule of wild-type mice with STZ-induced diabetes to construct an islets allograft transplantation model and to explore whether m6A modification affected the function of Treg cells after transplantation. Transplant tolerance is induced by treatment with anti-CD45RB & anti-CD40L monoclonal antibodies (mAbs), which are used to decrease TCR sensitivity and prevent graft rejection (26, 27) (**Supplementary Figure 1A**). Allograft rejection was defined as the two successive non-fasting blood glucose concentrations exceeding 11.1 mmol/L (400 mg/dL). In the absence of mAbs treatment (n=7), the islets allograft was rejected rapidly, with a mean survival time of approximately 13 ± 2.2 days. The mAbs treatment induced long-term graft survival of more than 90 days, which returned to a hyperglycemic state by nephrectomy. These results indicated that normoglycemia was achieved due to the functional islets graft (**Figures 1A, B**). Subsequently, Treg cells were isolated separately from the group of allograft tolerant mice (Treg-AT cells) or the group of allograft rejection mice (Treg-AR cells). The level of m6A in total RNA extracted from Treg-AT and Treg-AR cells was detected using dot blotting with an m6A-specific antibody. We observed a 40% to 50% reduction in the m6A level in Treg-AR cells, compared to that in Treg-AT cells (**Figures 1C, D**). Moreover, we examined the mRNA and protein levels of m6A regulators, including ‘writer’ enzymes (METTL3 and METTL14) and ‘eraser’ enzymes (ALKBH5 and FTO), in Treg-AT and Treg-AR cells. The expression of METTL3 and METTL14 was significantly lower in Treg-AR cells than in Treg-AT cells at both the mRNA and protein levels (**Figures 1E–G**). These results implied that m6A modifications were involved in maintaining functional Treg cells after transplantation, and may affect the induction of transplant tolerance.

### METTL14 deficient Treg cells failed to maintain islets allograft survival

**Figure 2:**
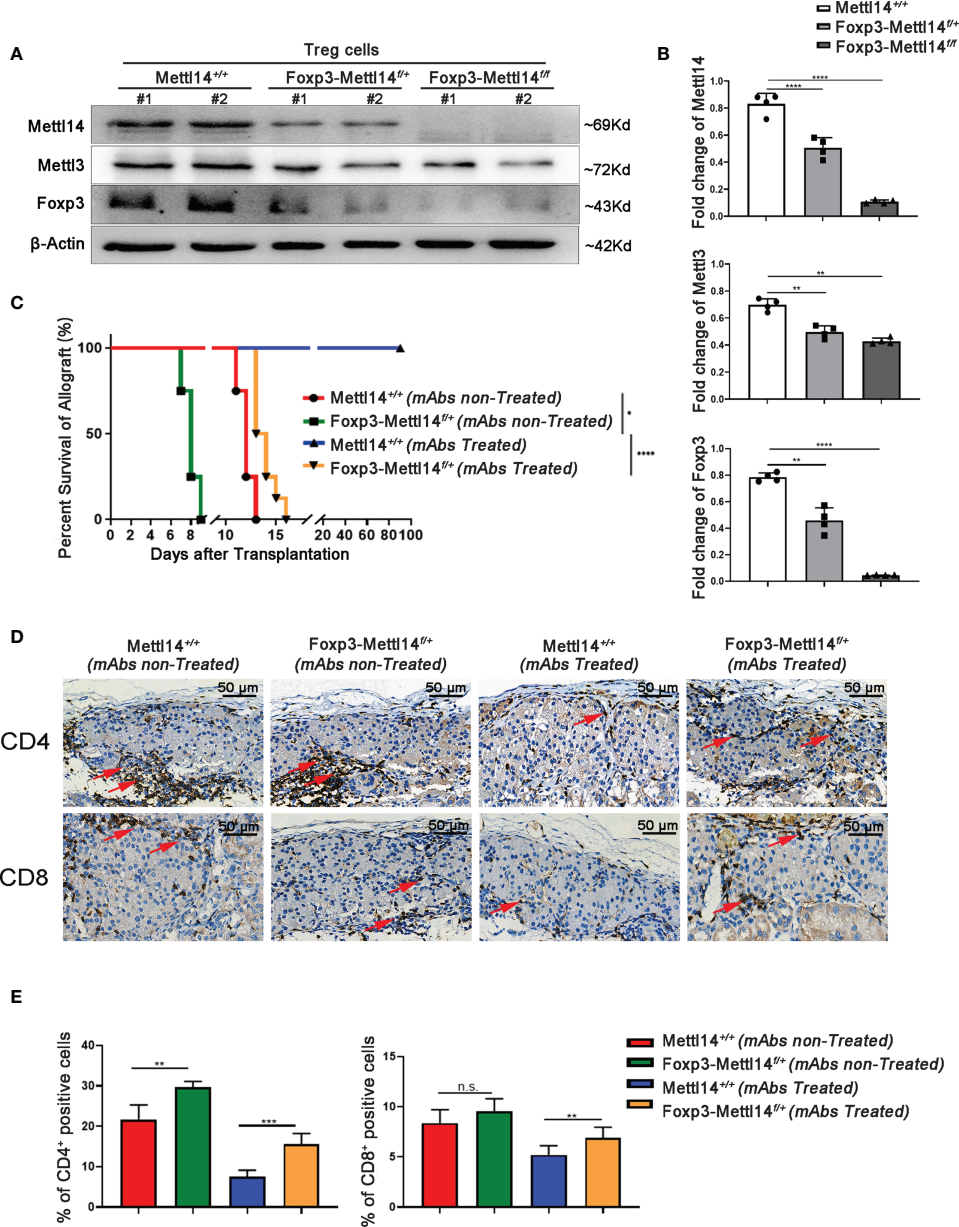
The METTL14-mediated m6A modification affected islet allograft tolerance. **(A, B)** Western blot analysis revealed that the expression of FOXP3, METTL14 and METTL3 in Treg cells isolation from Foxp3-Mettl14*^f/+^* and Foxp3-Mettl14*^f/f^* cKO mice was significantly decreased compared to the levels in Treg cells from littermate controls. **(C)** Without mAbs treatment, allografts were rejected in littermate controls (n=7, 14 ± 1.2, red) and Foxp3-Mettl14*^f/+^* cKO mice (n=7, 7.9 ± 1.8, green). With mAbs treatment, long-term graft survival (blue) was observed in littermate controls, while islets allograft was rejected in Foxp3-Mettl14*^f/+^* cKO mice (n=7, 15 ± 3.2, yellow). **(D, E)** Immunohistochemistry and the quantitation of the results revealed the infiltration of CD4^+^ and CD8^+^ T cells around islet allografts on day 7 post-transplantation (red arrow), indicating severe inflammation in this region. The images (40×) were captured; scale bar 50μm. The data are presents as the means ± SD and are representative of three separate experiments. The data in **(B)** depict the mean protein expression normalized to the gene β-Actin. The allograft survival analysis was performed with a log-rank test. The statistical analysis was performed with an unpaired Student’s t-test (two-tailed). ****P<0.0001, ***P<0.001 and **P<0.01. P>0.05 was considered nonsignificant (n.s.).

We first generated Treg-specific heterozygous (Foxp3-Mettl14^f/+^) and homozygous (Foxp3-Mettl14^f/f^) mice using the Cre-loxp system to assess whether the m6A modification in Treg cells affects allograft acceptance after transplantation (**Supplementary Figure 2A**). The Treg cells were separated from the Mettl14^f/f^, heterozygous (Foxp3-Mettl14^f/+^) and homozygous (Foxp3-Mettl14^f/f^) mice. The purity of Foxp3^+^ Treg cells is 90% ± 2.3%, 89% ± 1.2% and 90% ± 3.5% in Mettl14^f/f^, heterozygous (Foxp3-Mettl14^f/+^) and homozygous (Foxp3-Mettl14^f/f^) mice (**Supplementary Figure 2B**). In this animal model, the Mettl14 allele is selectively depleted or reduced in Treg cells, and the western blot analysis showed the protein level of METTL14 was significantly abrogated in Treg cells isolated from either heterozygotes or homozygotes. Meanwhile, the expression of METTL3, the core component of the RNA methyltransferase complex, was also reduced to some extent, similar to a previous report (28). Moreover, compared to the littermate controls, a 60% to 90% decrease in FOXP3 expression was observed in Treg cells from Foxp3-Mettl14^f/+^ and Foxp3-Mettl14^f/f^ cKO mice (**Figures 2A, B**).

Next, we explored the effect of METTL14-deficient Tregs on transplant tolerance induction by using an allograft transplantation model. However, homozygous Foxp3-Mettl14^f/f^ cKO mice developed a severe autoimmune disease and died within eight to twelve weeks, similar to the results for Mettl3-Treg cKO mice (11). We subsequently used heterozygous Foxp3-Mettl14^f/+^ cKO mice to perform islets transplantation, unless indicated otherwise stated. Consistent with our previous studies, stable long-term allograft survival was not established in either Foxp3-Mettl14^f/+^ cKO mice, or littermate controls in the absence of mAbs treatment, and the grafts were rejected within two weeks after transplantation. Despite the presence of mAbs, Foxp3-Mettl14^f/+^ cKO mice were not able to achieve long-term survival of the allograft. The euglycemia in Foxp3-Mettl14^f/+^ cKO mice was maintained for 15 ± 3.2 days, which was significantly reduced compared to the littermate controls (**Figure 2C**). As the deleterious inflammatory response around the graft is one of the main causes of rejection after transplantation, we next explored the infiltrated CD4^+^ and CD8^+^ T cells around the graft in Foxp3-Mettl14^f/+^ cKO mice and littermate controls on day 7 post-transplantation. The number of CD4^+^ and CD8^+^ T cells were significantly increased around the grafts in Foxp3-Mettl14^f/+^ cKO mice, regardless of the presence or absence of mAbs treatment (**Figures 2D, E**). These results suggested that the METTL14-mediated m6A modification in Treg cells plays a key role in long-term allograft survival post-transplantation.

### The population of Treg cells was limited in the Foxp3-Mettl14^f/+^ cKO mice after transplantation

**Figure 3:**
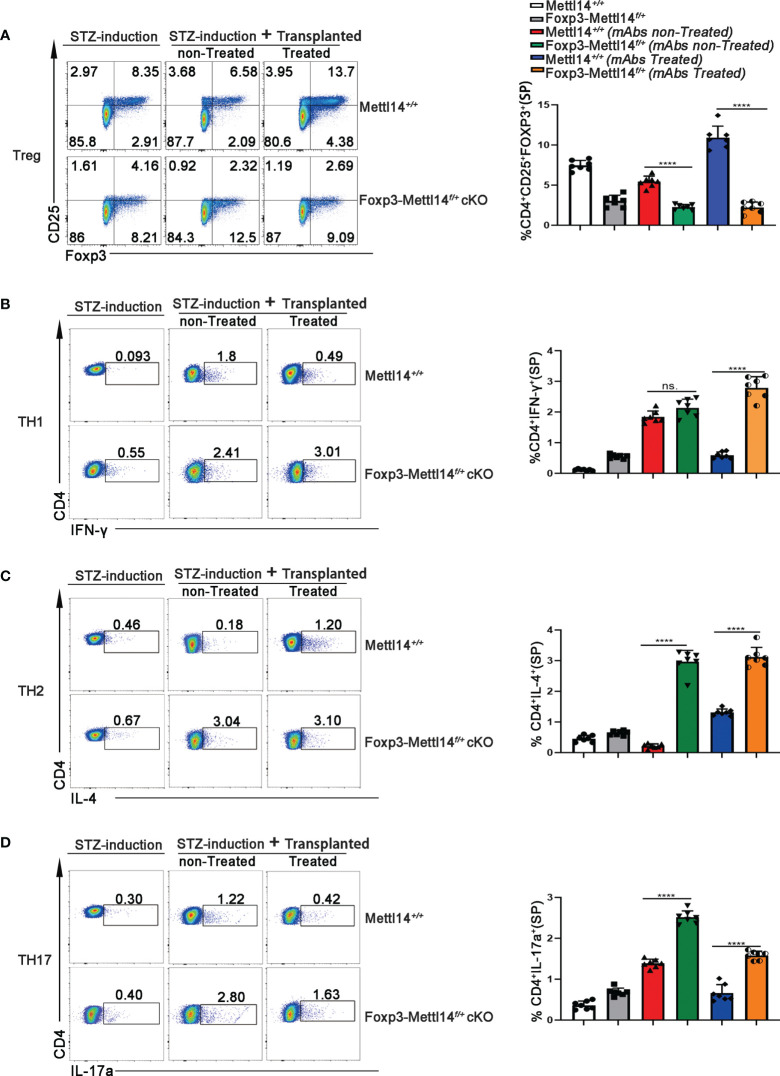
The METTL14 mediated m6A modification is needed to maintain the population of Treg cells post-transplantation. **(A)** Representative FACS analysis showing the proportion of Treg cells (CD4^+^CD25^+^Foxp3^+^) in among the CD4^+^ T-cell population in the spleens at day 7 after transplantation (n=7). In the presence or absence of mAbs, the proportion of CD4^+^CD25^+^Foxp3^+^ Treg cells was decreased in Foxp3-Mettl14*^f/+^* cKO mice compared to littermate controls after islets allograft transplantation. **(B–D)** Representative FACS analysis showing the proportion of TH1 (CD4^+^IFN-γ^+^TH1), TH2 (CD4^+^IL-4^+^TH2) and TH17 (CD4^+^IL-17a^+^TH17) cells among the CD4^+^ T-cell population in the spleen at day 7 after transplantation (n=7). Compared to littermate controls, the proportions of other main subtypes of T-cell including CD4^+^IFN-γ^+^TH1 **(B)**, CD4^+^IL-4^+^TH2 **(C)** and CD4^+^IL-17a^+^TH17 **(D)** were significantly increased in Foxp3-Mettl14*^f/+^* cKO mice after islets allograft transplantation. The data are shown as the means ± SD, the statistical analysis was performed with an unpaired Student’s t-test (two-tailed), ****P<0.0001. P>0.05 was considered nonsignificant (n.s.).

The proportion of Treg cells is critical in controlling immune homeostasis and tolerance after transplantation (29). Therefore, we analyzed the percentage of CD4^+^CD25^+^FOXP3^+^Treg cells, from spleen and renal draining lymph nodes (RDLNs), on day 7 post-transplantation in Foxp3-Mettl14^f/+^ cKO mice and littermate controls. Without mAbs treatment, the level of the Foxp3^+^ population decreased significantly in Foxp3-Mettl14^f/+^ cKO mice, compared to the littermate controls. However, with treatment of anti-CD45RB and anti-CD40L, the level of Foxp3 maintain low population in the Foxp3-Mettl14^f/+^ cKO mice after transplantation (**Figure 3A** and **Supplementary Figure 3A**). These data suggested that the METTL14-mediated m6A modification in Treg cells is important for preserving and expansion the number of Treg cells after transplantation.

Since CD4^+^ T cells function as helper subsets (TH1, TH2 and TH17) after transplantation to generate specific cytokine profiles, resulting in graft damage (30, 31), we also analyzed the ratios of TH1, TH2, and TH17 in Foxp3-Mettl14^f/+^ cKO mice and littermate controls receiving identical treatments post-transplantation using flow cytometry. We observed significant increases in the TH1, TH2, and TH17 populations in Foxp3-Mettl14^f/+^ cKO mice compared with those in littermate controls mice (**Figures 3B–D** and **Supplementary Figure 3B-3D**). Thus, the METTL14 deficiency Treg cells inhibited functional Treg cells expansion and resulted in TH1, TH2, and TH17 polarization, which may ultimately lead to transplant rejection.

### The METTL14 mediated m6A modification is required for the suppressive function of Treg cells after transplantation

**Figure 4:**
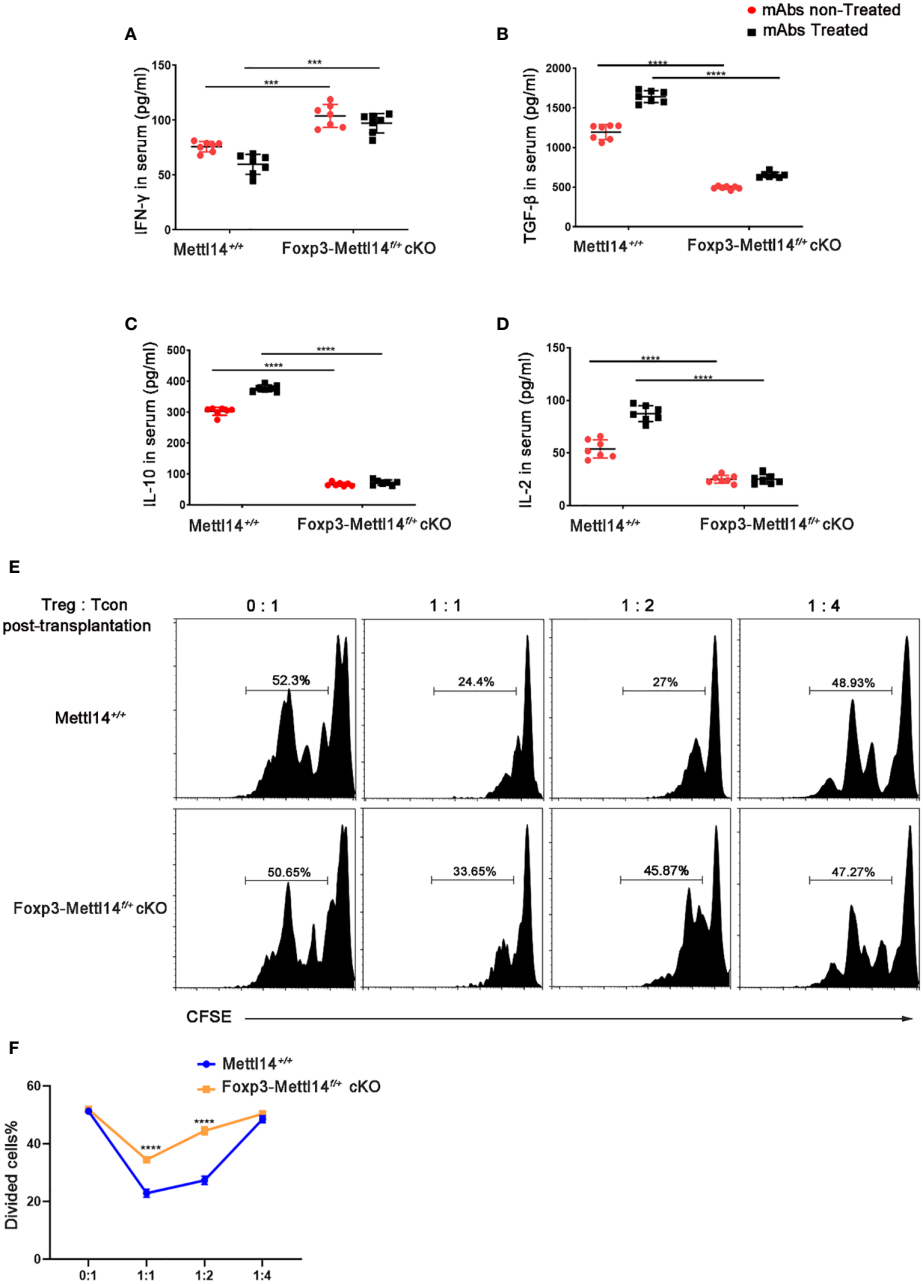
METTL14 deficiency Treg cells resulted in the loss of suppressive function after transplantation. **(A–D)** Serum was collected on day 7 after transplantation and cytokine levels were measured using ELISA assay. In the presence or absence of mAbs treatment, the scatter plots show the mean serum cytokine concentration in picograms per milliliter of IFN-γ **(A)**, TGF-β **(B)**, IL-10 **(C)** and IL-2 **(D)** from serum in Foxp3-Mettl14f/+ cKO mice and littermate controls (n=7). **(E)** The islets were isolated from BALB/c mice and transplanted them under the kidney capsule of littermate controls or Foxp3-Mettl14f/+ cKO mice with STZ-induced diabetes. These mice were treated with mAbs as described above. Treg cells were isolated on day 7 post-transplantation which were co-cultured with CFSE-labeled CD4+ naïve T cells from C57BL/6J mice, in round-bottom 96-well plates containing anti-CD3 (3 μg/ml) and anti-CD28 (5 μg/ml) monoclonal antibodies, at various ratios for 5 days. The suppressive effect on T cell expansion was detected using flow cytometry. **(F)** The suppressive function of Treg cells was quantified. The data are presented as the means ± SD. The data in **(A-D)** depict the mean values measured from seven separate experiments, while the data in **(D)** are representative of at least three independent experiments. The statistical analysis was performed with an unpaired Student’s t-test (two-tailed).

Suppressive factors, such as TGF-β, IL-2 and IL-10, are critical regulator of the function and development in Treg cells, the proinflammatory cytokine IFN-γ is negatively related to functional Treg cells (32). We evaluated the changes in cytokine levels triggered in Foxp3-Mettl14^f/+^ cKO mice after transplantation and observed alterations. Specifically, the level of the effector cytokines IFN-γ was elevated, while the levels of the functional Treg-associated factors TGF-β, and IL-10 and IL-2 were significantly decreased in Foxp3-Mettl14^f/+^ cKO mice, compared to littermate controls after transplantation (**Figures 4A–D**).

Previous studies revealed that proper control of the CD45RB level might promote the expansion of functional Treg cells, which is why researchers have used the anti-CD45RB antibody in transplantation (33). However, in our study, the Foxp3-Mettl14*^f/+^* cKO mice rapidly rejected the islets allograft even while receiving mAbs treatment compared to the littermate controls. We thus hypothesized that the METTL14-mediated m6A modification is involved in the function of Treg cells after transplantation. We established a co-culture system of Treg cells with CFSE labeled-CD4^+^ naïve T cells (T-con) at various ratios *ex vivo* to confirm the effect of METTL14 deficiency on the suppressive function of Treg cells after transplantation. Treg cells were isolated from Foxp3-Mettl14^f/+^ mice or littermate controls on day 7 post-transplantation. According to the suppressive T-cell proliferation assay, we found that Treg cells isolated from Foxp3-Mettl14^f/+^ mice exhibited a remarkably decreased ability to suppress naïve T-cell proliferation, regardless of the ratio compared to the Treg cells isolated from the littermate controls (**Figures 4E, F**).

### The loss of METTL14 disrupted the stability of functional Treg cells *in vitro*

**Figure 5:**
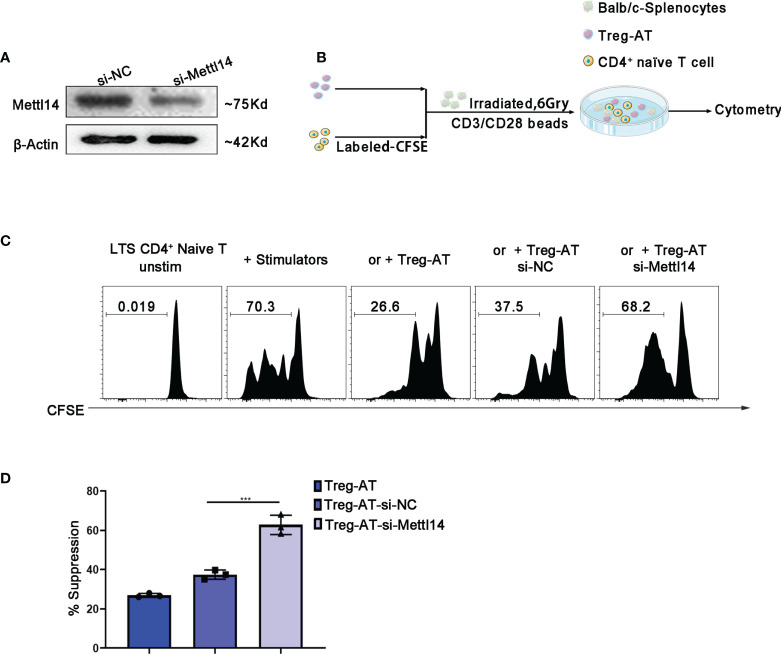
Manipulation of functional Treg cells by disturbing METTL14 expression *in vitro.* **(A)** Treg cells isolated from the group of allograft tolerance (Treg-AT), and transfected with si-negative control (Treg-AT-si-NC) or si-Mettl14 (Treg-AT-si-Mettl14). METTL14 expression was detected using western blot. **(B)** The experimental plan for the mixed lymphocyte response assay. In briefly, the CD4^+^ naïve T-cells (0.3×10^6^) serving as responders were isolated from the spleen and labeled with CFSE. The BALB/c splenocytes (2×10^5^) were irradiated (6 Gry) and served as allogeneic stimulator cells. In general, the responder cells and stimulator cells were resuspended in 96-well round-bottom plates, and T-activator CD3/CD28 Dynabeads (0.5μg/ml) were added. Subsequently, the Treg-AT, Treg-AT-si-NC or Treg-AT-si-Mettl14 (0.2×10^4^) was added to each well. These cells were cultured at 37°C for 5 days, and proliferation was monitored using flow cytometry. **(C)** Mixed lymphocyte response assays were performed to determine the suppressive function and stability of Treg cells in the allograft tolerance group. CFSE-labeled CD4^+^ naïve T cells as responder cells, and were stimulated with BALB/c splenocytes, and the Treg-AT, Treg-AT-si-NC, or Treg-AT-si-Mettl14 were used as suppressor cells in this assay. The ratio of proliferation CD4^+^ naïve T cells in the presence of Treg-AT, Treg-AT-si-NC, or Treg-AT-si-Mettl14 was detected using flow cytometry. The data are presented as the means ± SD. The data **(D)** are representative of three independent experiments. The statistical analysis was performed with an unpaired Student’s t-test (two-tailed). ***P<0.001.

Next, we decided to further confirm the importance of the m6A modification to the functional Treg cells, which were isolated from the group of allograft tolerance mice (Treg-AT cells). It was suggested to have a strong suppressive function in our previous studies (34). We isolated Treg cells from the allograft tolerance group after transplantation and transfected cells with a siRNA to specifically knockdown METTL14 expression (**Figure 5A**). Subsequently, we performed a mixed lymphocyte reaction assay to determine the suppressive effect of Treg-AT cells after METTL14 knockdown (**Figure 5B**). In the absence of Treg cells, sensitized CD4^+^ naïve T cells rapidly responded to stimulators, originating from BALB/c mice, and proliferated actively. However, METTL14 knockdown significantly abrogated the suppressive function of Treg-AT cells and resulted in an increased proliferation rate of CD4^+^ naïve T cells in response to the alloantigen (**Figures 5C, D**). Thus, the METTL14-mediated m6A modification is required to maintain the stability of functional Treg cells after transplantation.

### Signaling pathway affected in Foxp3-Mettl14^f/+^ cKO mice after transplantation

**Figure 6:**
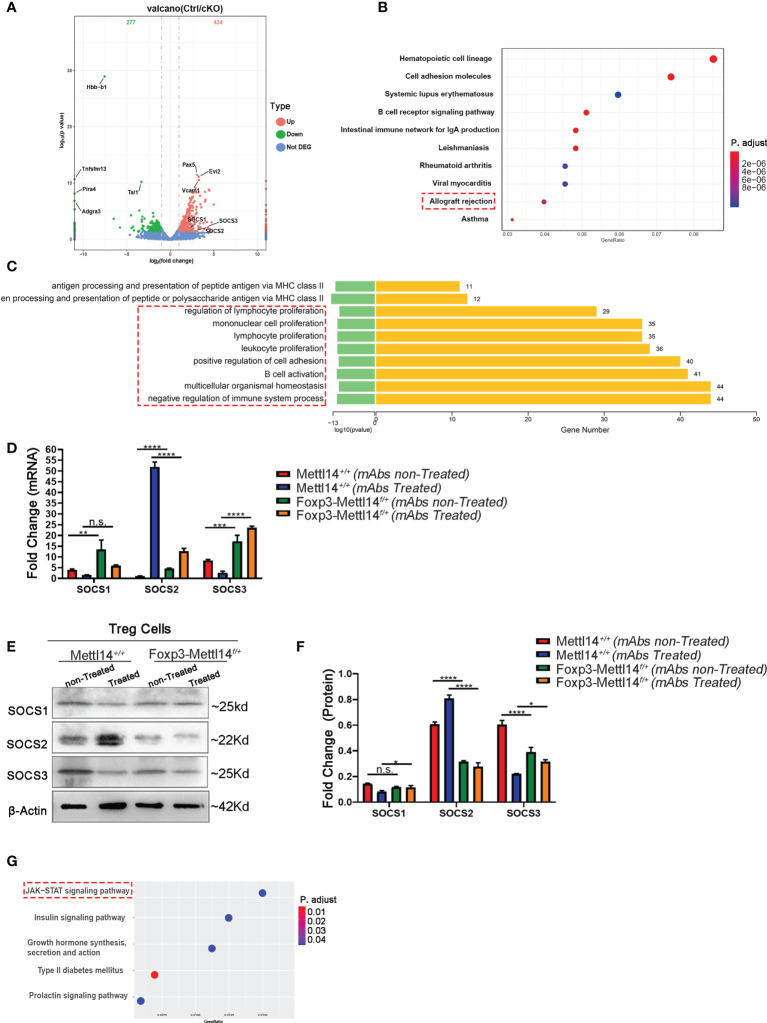
The SOCS family was disturbed in Foxp3-Mettl14*^f/+^* cKO mice. **(A)** RNA-seq indicated that 434 genes were upregulated and 277 genes were downregulated in Treg cells from Foxp3-Mettl14*^f/+^* cKO mice compared with the littermate controls. **(B)** Gene Ontology (GO) enrichment analysis indicated that transplantation related signaling pathways were altered in Treg cells from Foxp3-Mettl14*^f/+^* cKO mice. **(C)** KEGG pathways were enriched in proliferation and activation in immune cells. **(D–F)** The expression of SOCS1, SOCS2 and SOCS3 in Treg cells from littermate controls and Foxp3-Mettl14*^f/+^* cKO mice on day 7 post-transplantation in the presence or absence of mAbs was validated using qPCR and western blotting. **(G)** KEGG pathway enrichment analysis of the RNA-seq results for the Socs2 gene, which indicated that the JAK-STAT signaling pathway was altered in METTL14 deficient Treg cells post-transplantation. The data are shown as the means ± SD and are representative of three separate experiments. The data in **(D)** depict the mean mRNA expression normalized to the gene Gapdh. The data in **(F)** depict the mean protein expression normalized to the β-Actin.The statistical analysis was performed with an unpaired Student’s t-test (two-tailed).****P<0.0001, ***P<0.001, **P<0.01, *P<0.05. *P* values >0.05 were considered nonsignificant (n.s.).

We further investigated the effect of the METTL14 mediated m6A modification on the transcriptome of Treg cells after transplantation. We performed an RNA sequencing (RNA-seq) analysis of Treg cells, which were isolated from Foxp3-Mettl14^f/+^ cKO mice and the littermate controls on day 7 post-transplantation without mAbs treatment. The transcripts levels of 434 genes were upregulated and those of 277 genes were downregulated in Treg cells from Foxp3-Mettl14^f/+^ cKO mice compared to those genes in Treg cells from the littermate controls (**Figure 6A**, **Supplementary Table 1**). Moreover, the KEGG pathway enrichment analysis revealed that the differentially expressed genes in Treg cells lacking METTL14 is mainly related to ‘allograft rejection’ and ‘immunoinflammatory diseases’ (**Figure 6B**). Additionally, the GO enrichment analysis indicated that the biological processes related to lymphocyte differentiation and activation were significantly altered in the absence of METTL14 (**Figure 6C**). Among them, suppressors of cytokine signaling (SOCS), a specialized family of proteins were upregulated in Treg cells from Foxp3-Mettl14f/+ cKO mice after transplantation (**Figure 6A**). The SOCS family is known to participate in the negative feedback regulation of cytokine signaling to maintain appropriate immune cell development and function. Moreover, it is also involved in the regulation of cytokine receptor signaling which affects the processes of inflammation and transplant tolerance (35–37). In our study, consistent with previous findings from Mettl3-Treg cKO mice (11), the mRNA levels of SOCS1, SOCS2 and SOCS3 in Treg cells were increased in Foxp3-Mettl14^f/+^ cKO mice post-transplantation without mAbs treatment (**Figure 6D**). However, the levels of SOCS2 and SOCS3 (particularly SOCS2) were decreased, and no changes in SOCS1, in Treg cells at the translational levels, compared to those in Treg cells isolated from littermate controls (**Figures 6E, F**). Furthermore, the KEGG pathway analysis indicated that the SOCS2 expression was mainly related to the JAK-STAT signaling pathway, which is closely related to the differentiation and function of Treg cells (38) (**Figure 6G**). Thus, these results indicated that the METTL14-mediated m6A modification might affect the expression and relative signaling of the SOCS family, especially SOCS2, in Treg cells after transplantation and it may further impact the outcome of transplant tolerance.

## Discussion

Among post-transcriptional modifications, m6A is the most abundant mRNA modification in mammals. It plays an important role in the translational control to regulate immune cells (including DCs, T cells, B cells, and Treg cells) within cancer and inflammatory diseases. However, the requirement of m6A modification for Treg cells in transplantation has not been reported. In the present study, we showed that the methyltransferase METTL14 is necessary for allograft acceptance. Meanwhile, we found that that METTL14-mediated m6A modification is essential for the stability, expansion and function of Treg cells post-transplantation, which was probably mediated by the translational regulation of SOCS family proteins. Our findings demonstrated for the first time the regulatory function of the m6A modification in transplant medicine.

FOXP3 is essential for the development and suppressive function of Tregs, and it also plays a key role in maintaining the immune homeostasis. Loss or disruption of stable FOXP3 expression results in overt lymphoproliferative disease, autoimmunity, and graft rejection (39–41). It has been reported that the epigenetic modification including DNA methylation, histone modification, and chromatin remodeling play a role in FOXP3 expression (42). Moreover, RNA can also be modified, which serves as a key biomarker for several biological events. The N6-methyladenosine (m6A) is the most abundant mRNA modification in mammals to regulate mRNA processing and metabolism (export, splicing, decay, translation) (43, 44). It has been reported that the lineage-specific deletion of Mettl3 in Treg cells leads to the loss of Foxp3-dependent transcriptional activation and gene expression which contributes to the development of autoimmunity disease and subsequent death in the Foxp3-Mettl3^f/f^ cKO mice (11, 40), similar to what was observed in the Mettl14^f/f^ cKO mice. Therefore, METTL14-mediated m6A modification is required to maintain the continuous expression of Foxp3 and keep the suppressive function of Treg cells. Besides, it was also reported that METTL14 deficient in CD4^+^ T cells leads to increased infiltration of inflammatory cells with induced pro-inflammatory cytokines. Meanwhile, inhibition of the development from naïve T cells to induced Treg cells was also observed in that study (12). Our results lead to similar conclusion where the function and stability of Treg cells in Foxp3-Mettl14^f/+^ cKO mice were significantly inhibited (**Figure 4D**). Furthermore, Treg cells isolated from the group of allograft tolerance lost the function to inhibit the proliferation of naïve T cells in the absence of METTL14 (**Figure 5C**). In addition, we also found the infiltration of immune cells (CD4^+^ and CD8^+^) increased around the allograft in Foxp3-Mettl14^f/+^ cKO mice, which might be a direct cause of the allograft rejection in our study (11). Meanwhile, in line with our studies that specific deletion of Mettl14 in CD4^+^ T cells has also been shown to cause spontaneous colitis in mice, which is the outcome of T cell dysfunction and subsequent loss of the inhibitory activity of Treg cells (12). These results suggested that METTL14-mediated m6A modification is involved in the expression of FOXP3, which further affect the stability and function of Treg cells after transplantation.

The suppressor of cytokine signaling proteins (SOCS) family consists of intracellular proteins that regulate the development of Treg cells by controlling cytokine secretion. Meanwhile, the SOCS family of proteins plays a key role in the metabolic and inflammatory response of immune cells (45, 46). METTL3 deficiency in CD4^+^ T cells leads to aberrant differentiation of Treg cells, which is caused by increased SOCS levels and inhibition of the IL-2-STAT5 signaling pathway (47). Furthermore, SOCS1 and SOCS3 proteins were shown to regulate Treg cells function by specifically disrupting the generation of IL-10 and TGF-β (48). In our study, we found that the SOCS family was upregulated in Foxp3-Mettl14^f/+^ cKO mice by using high-throughput RNA-Seq and qPCR analysis. However, the protein expression of SOCS2, SOCS3 decreased in Foxp3-Mettl14*^f/+^* cKO mice compared to those in littermate controls (**Figures 6E, F**). Previous studies have revealed that SOCS2 is highly expressed in induced Treg (iTreg) cells, and deletion of SOCS2 selectively affect the stability of iTreg cells (49). Combined with our findings, we speculated that the regulation of the SOCS family by m6A modification acts as a “valve”, which is closely related to the stability and function of Treg cells after transplantation; thus, the underlying mechanism is needed for further study.

According to previous studies, depletion of Foxp3^+^ Treg cells by anti-CD25 mAb after transplantation leads to the subversion of transplant tolerance to rejection in animal models (50). Preclinical evidence suggests that the use of Treg cells therapy is effective in diminishing the usage of immunosuppressive drugs and preventing allograft rejection (51). In our study, we found that the METTL14 deficient Treg cells inhibited its suppressive function by disrupting the expression of FOXP3. Our study suggested that it might be a novel marker to check the functional Tregs by monitoring the level of m6A modification after transplantation. In clinical, it is a novel strategy to transfer adoptive Treg cells in order to preserve the number of functional Treg cells post-transplantation for induction of immune tolerance. However, the main obstacle of adoptive Treg cells therapy is the unstable property of Treg cells *in vivo* (52). Therefore, harnessing the natural power of epigenetic modification to enhance the stability and number of functional Treg cells could provide alternative strategy to improve the allograft acceptance. Since our work underscores the significance of epitranscriptomic modification for the expression of FOXP3 as well as the development of Treg cells, it is exciting to expect that the development of pharmacological agonist or inhibitors to modulate the m6A related enzymes could be a promising strategy for improving the suppressive function of transferred Treg cells post-transplantation.

Taken together, our study for the first to explored the significance of m6A modification in Treg cells post-transplantation. As functional Treg cells are recognized as one of the most promising cell-based therapies for the induction of immune tolerance in allogenic transplantation, researchers have attempted to explore appropriate strategies to modulate and improve the function of Treg cells as methods to benefit long-term transplant outcomes. Our results also suggested that modulation of m6A might be a potential regulatory strategy to control the development, differentiation, and function of Treg cells post-transplantation and it provided fundamental research data to explore the underlying molecular mechanisms involved in m6A modification mediated Treg cell function in the future.

## Data availability statement

The data presented in the study are deposited in the GEO repository, accession number GSE215371.

## Ethics statement

The animal procedures were compliant with the Institutional Animal Care and Use Committee of the University of Electronic Science and Technology of China.

## Author contributions

GZ and LY contributed to research concept, research administration and support. YL carried out the experiments with the help of YY, YC, YT, HY, YZ and HH analyzed the data. GZ, LY, and YL wrote the manuscript. All authors edited and approved the final version of the manuscript.

## Funding

This work was supported by funding from the National Natural Science Foundation of China (81771723), the Department of Science and Technology of Sichuan Province Grant numbers (22ZDYF2062), and the Department of Science and Technology of Sichuan Province Grant numbers (2021ZYD0093, 2022YFS0597, 22ZDYF2062).

## Acknowledgments

The authors would like to thank Dr. Li Hua-bing (Shanghai Institute of Immunology, State Key Laboratory of Oncogenes and Related Genes, School of Medicine, Shanghai Jiao Tong University Shanghai 200025, China) for providing the Mettl14-loxp mice.

## Conflict of interest

The authors declare that the research was conducted in the absence of any commercial or financial relationships that could be construed as a potential conflict of interest.

## Publisher’s note

All claims expressed in this article are solely those of the authors and do not necessarily represent those of their affiliated organizations, or those of the publisher, the editors and the reviewers. Any product that may be evaluated in this article, or claim that may be made by its manufacturer, is not guaranteed or endorsed by the publisher.

## References

[1] Garner L. Distance from a transplant center and getting listed for a transplant. Clin J Am Soc Nephrol: CJASN (2020) 15(4):439–40. doi: 10.2215/CJN.02130220 PMC7133137 32273260

[2] Ingulli E. Mechanism of cellular rejection in transplantation. Pediatr Nephrol (Berlin Germany) (2010) 25(1):61–74. doi: 10.1007/s00467-008-1020-x PMC2778785 21476231

[3] Cao P Sun Z Feng C Zhang J Zhang F Wang W. Myeloid-derived suppressor cells in transplantation tolerance induction. Int Immunopharmacol (2020) 83:106421. doi: 10.1016/j.intimp.2020.106421 32217462

[4] Lechler RI Garden OA Turka LA. The complementary roles of deletion and regulation in transplantation tolerance. Nat Rev Immunol (2003) 3(2):147–58. doi: 10.1038/nri1002 12563298

[5] Cobbold SP Adams E Graca L Daley S Yates S Paterson A. Immune privilege induced by regulatory T cells in transplantation tolerance. Immunol Rev (2006) 213:239–55. doi: 10.1111/j.1600-065X.2006.00428.x 16972908

[6] Haberle V Stark A. Eukaryotic core promoters and the functional basis of transcription initiation. Nat Rev Mol Cell Biol (2018) 19(10):621–37. doi: 10.1038/s41580-018-0028-8 PMC6205604 29946135

[7] Oerum S Meynier V Catala M Tisné C. A comprehensive review of m6A/m6Am RNA methyltransferase structures. Nucleic Acids Res (2021) 49(13):7239–55. doi: 10.1093/nar/gkab378 PMC8287941 34023900

[8] Zhao BS Roundtree IA He C. Post-transcriptional gene regulation by mRNA modifications. Nat Rev Mol Cell Biol (2017) 18(1):31–42. doi: 10.1038/nrm.2016.132 27808276 PMC5167638

[9] Fu Y Dominissini D Rechavi G He C. Gene expression regulation mediated through reversible m^6^A RNA methylation. Nat Rev Genet (2014) 15(5):293–306. doi: 10.1038/nrg3724 24662220

[10] Vu LP Pickering BF Cheng Y Zaccara S Nguyen D Minuesa G. The N(6)-methyladenosine (m(6)A)-forming enzyme METTL3 controls myeloid differentiation of normal hematopoietic and leukemia cells. Nat Med (2017) 23(11):1369–76. doi: 10.1038/nm.4416 PMC5677536 28920958

[11] Tong J Cao G Zhang T Sefik E Amezcua Vesely MC Broughton JP. m(6)A mRNA methylation sustains treg suppressive functions. Cell Res (2018) 28(2):253–6. doi: 10.1038/cr.2018.7 PMC5799823 29303144

[12] Lu TX Zheng Z Zhang L Sun HL Bissonnette M Huang H. A new model of spontaneous colitis in mice induced by deletion of an RNA m(6)A methyltransferase component METTL14 in T cells. Cell Mol Gastroenterol Hepatol (2020) 10(4):747–61. doi: 10.1016/j.jcmgh.2020.07.001 PMC7498954 32634481

[13] Raffin C Vo LT Bluestone JA. T(reg) cell-based therapies: challenges and perspectives. Nat Rev Immunol (2020) 20(3):158–72. doi: 10.1038/s41577-019-0232-6 PMC7814338 31811270

[14] Lee KM Stott RT Zhao G SooHoo J Xiong W Lian MM. TGF-β-producing regulatory b cells induce regulatory T cells and promote transplantation tolerance. Eur J Immunol (2014) 44(6):1728–36. doi: 10.1002/eji.201344062 PMC4048633 24700192

[15] Mahato RI. Gene expression and silencing for improved islet transplantation. J Controlled Release: Off J Controlled Release Society (2009) 140(3):262–7. doi: 10.1016/j.jconrel.2009.04.011 PMC2933060 19376168

[16] Fallarino F Grohmann U Hwang KW Orabona C Vacca C Bianchi R. Modulation of tryptophan catabolism by regulatory T cells. Nat Immunol (2003) 4(12):1206–12. doi: 10.1038/ni1003 14578884

[17] Qu Y Zhang B Liu S Zhang A Wu T Zhao Y. 2-gy whole-body irradiation significantly alters the balance of CD4+ CD25- T effector cells and CD4+ CD25+ Foxp3+ T regulatory cells in mice. Cell Mol Immunol (2010) 7(6):419–27. doi: 10.1038/cmi.2010.45 PMC4002961 20871628

[18] Yi FS Zhang X Zhai K Huang ZY Wu XZ Wu MT. TSAd plays a major role in Myo9b-mediated suppression of malignant pleural effusion by regulating T(H)1/T(H)17 cell response. J Immunol (Baltimore Md: 1950) (2020) 205(10):2926–35. doi: 10.4049/jimmunol.2000307 33046503

[19] Zhao G Moore DJ Kim JI Lee KM O’Connor M Yang M. An immunosufficient murine model for the study of human islets. Xenotransplantation (2014) 21(6):567–73. doi: 10.1111/xen.12126 PMC4262653 25041432

[20] Kim EY Lee EN Lee J Park HJ Chang CY Jung DY. Two-signal blockade with anti-CD45RB and anti-CD154 monoclonal antibodies inhibits graft rejection *via* CD4-dependent mechanisms in allogeneic skin transplantation. Exp Mol Med (2006) 38(3):284–94. doi: 10.1038/emm.2006.34 16819287

[21] Li Y Ding X Tian X Zheng J Ding C Li X. Islet transplantation modulates macrophage to induce immune tolerance and angiogenesis of islet tissue in type I diabetes mice model. Aging (2020) 12(23):24023–32. doi: 10.18632/aging.104085 PMC7762494 33221752

[22] Piao W Xiong Y Li L Saxena V Smith KD Hippen KL. Regulatory T cells condition lymphatic endothelia for enhanced transendothelial migration. Cell Rep (2020) 30(4):1052–62.e5. doi: 10.1016/j.celrep.2019.12.083 31995749 PMC7009789

[23] Xiong Y Wang Y Zhang J Zhao N Zhang H Zhang A. hPMSCs protects against d-galactose-induced oxidative damage of CD4(+) T cells through activating akt-mediated Nrf2 antioxidant signaling. Stem Cell Res Ther (2020) 11(1):468. doi: 10.1186/s13287-020-01993-0 33148324 PMC7641865

[24] Cheung AS Zhang DKY Koshy ST Mooney DJ. Scaffolds that mimic antigen-presenting cells enable ex vivo expansion of primary T cells. Nat Biotechnol (2018) 36(2):160–9. doi: 10.1038/nbt.4047 PMC5801009 29334370

[25] Grosjean C Quessada J Nozais M Loosveld M Payet-Bornet D Mionnet C. Isolation and enrichment of mouse splenic T cells for ex vivo and *in vivo* T cell receptor stimulation assays. STAR Protoc (2021) 2(4):100961. doi: 10.1016/j.xpro.2021.100961 34825221 PMC8605083

[26] Koretzky GA Picus J Thomas ML Weiss A. Tyrosine phosphatase CD45 is essential for coupling T-cell antigen receptor to the phosphatidyl inositol pathway. Nature (1990) 346(6279):66–8. doi: 10.1038/346066a0 2164155

[27] Deng S Moore DJ Huang X Mohiuddin M MKt L Velidedeoglu E. Antibody-induced transplantation tolerance that is dependent on thymus-derived regulatory T cells. J Immunol (Baltimore Md: 1950) (2006) 176(5):2799–807. doi: 10.4049/jimmunol.176.5.2799 16493036

[28] Yang Y Shuai P Li X Sun K Jiang X Liu W. Mettl14-mediated m6A modification is essential for visual function and retinal photoreceptor survival. BMC Biol (2022) 20(1):140. doi: 10.1186/s12915-022-01335-x 35698136 PMC9195452

[29] Camirand G Riella LV. Treg-centric view of immunosuppressive drugs in transplantation: A balancing act. Am J Transplant: Off J Am Soc Transplant Am Soc Transplant Surgeons (2017) 17(3):601–10. doi: 10.1111/ajt.14029 27581661

[30] Burrell BE Bishop DK. Th17 cells and transplant acceptance. Transplantation (2010) 90(9):945–8. doi: 10.1097/TP.0b013e3181f5c3de PMC3191917 20838278

[31] Liu Z Fan H Jiang S. CD4(+) T-cell subsets in transplantation. Immunol Rev (2013) 252(1):183–91. doi: 10.1111/imr.12038 23405905

[32] Regateiro FS Howie D Cobbold SP Waldmann H. TGF-β in transplantation tolerance. Curr Opin Immunol (2011) 23(5):660–9. doi: 10.1016/j.coi.2011.07.003 21839624

[33] Kamanaka M Huber S Zenewicz LA Gagliani N Rathinam C O’Connor W Jr.. Memory/effector (CD45RB(lo)) CD4 T cells are controlled directly by IL-10 and cause IL-22-dependent intestinal pathology. J Exp Med (2011) 208(5):1027–40. doi: 10.1084/jem.20102149 PMC3092344 21518800

[34] Zhao G Moore DJ Kim JI Lee KM O’Connor MR Duff PE. Inhibition of transplantation tolerance by immune senescence is reversed by endocrine modulation. Sci Trans Med (2011) 3(87):87ra52. doi: 10.1126/scitranslmed.3002270 PMC3767303 21677198

[35] Silva LEF Lourenço JD Silva KR Santana FPR Kohler JB Moreira AR. Th17/Treg imbalance in COPD development: suppressors of cytokine signaling and signal transducers and activators of transcription proteins. Sci Rep (2020) 10(1):15287. doi: 10.1038/s41598-020-72305-y 32943702 PMC7499180

[36] Muthukumarana P Chae WJ Maher S Rosengard BR Bothwell AL Metcalfe SM. Regulatory transplantation tolerance and “stemness”: evidence that Foxp3 may play a regulatory role in SOCS-3 gene transcription. Transplantation (2007) 84(1 Suppl):S6–11. doi: 10.1097/01.tp.0000269116.06510.db 17632414

[37] de Weger RA. Immune regulators regulated to prevent transplant reactions. J Am Coll Cardiol (2014) 63(1):30–2. doi: 10.1016/j.jacc.2013.07.073 23994414

[38] Coskun M Salem M Pedersen J Nielsen OH. Involvement of JAK/STAT signaling in the pathogenesis of inflammatory bowel disease. Pharmacol Res (2013) 76:1–8. doi: 10.1016/j.phrs.2013.06.007 23827161

[39] Hori S Nomura T Sakaguchi S. Control of regulatory T cell development by the transcription factor Foxp3. Sci (New York NY) (2003) 299(5609):1057–61. doi: 10.1126/science.1079490 12522256

[40] Williams LM Rudensky AY. Maintenance of the Foxp3-dependent developmental program in mature regulatory T cells requires continued expression of Foxp3. Nat Immunol (2007) 8(3):277–84. doi: 10.1038/ni1437 17220892

[41] Wan YY Flavell RA. Regulatory T-cell functions are subverted and converted owing to attenuated Foxp3 expression. Nature (2007) 445(7129):766–70. doi: 10.1038/nature05479 17220876

[42] Ohkura N Sakaguchi S. Transcriptional and epigenetic basis of treg cell development and function: its genetic anomalies or variations in autoimmune diseases. Cell Res (2020) 30(6):465–74. doi: 10.1038/s41422-020-0324-7 PMC7264322 32367041

[43] Song T Yang Y Jiang S Peng J. Novel insights into adipogenesis from the perspective of transcriptional and RNA N6-Methyladenosine-Mediated post-transcriptional regulation. Advanced Sci (Weinheim Baden Wurttemberg Germany) (2020) 7(21):2001563. doi: 10.1002/advs.202001563 PMC7610318 33173729

[44] Wu J Frazier K Zhang J Gan Z Wang T Zhong X. Emerging role of m(6) a RNA methylation in nutritional physiology and metabolism. Obes Reviews: an Off J Int Assoc Study Obes (2020) 21(1):e12942. doi: 10.1111/obr.12942 PMC7427634 31475777

[45] Secombes C. Will advances in fish immunology change vaccination strategies? Fish Shellfish Immunol (2008) 25(4):409–16. doi: 10.1016/j.fsi.2008.05.001 18562212

[46] Durham GA Williams JJL Nasim MT Palmer TM. Targeting SOCS proteins to control JAK-STAT signalling in disease. Trends Pharmacol Sci (2019) 40(5):298–308. doi: 10.1016/j.tips.2019.03.001 30948191

[47] Li HB Tong J Zhu S Batista PJ Duffy EE Zhao J. m(6)A mRNA methylation controls T cell homeostasis by targeting the IL-7/STAT5/SOCS pathways. Nature (2017) 548(7667):338–42. doi: 10.1038/nature23450 PMC5729908 28792938

[48] Kanti Ghosh A Sinha D Mukherjee S Biswas R Biswas T. IL-15 temporally reorients IL-10 biased b-1a cells toward IL-12 expression. Cell Mol Immunol (2016) 13(2):229–39. doi: 10.1038/cmi.2015.08 PMC4786629 25748019

[49] Knosp CA Schiering C Spence S Carroll HP Nel HJ Osbourn M. Regulation of Foxp3+ inducible regulatory T cell stability by SOCS2. J Immunol (Baltimore Md: 1950) (2013) 190(7):3235–45. doi: 10.4049/jimmunol.1201396 PMC3607399 23455506

[50] Huss DJ Pellerin AF Collette BP Kannan AK Peng L Datta A. Anti-CD25 monoclonal antibody fc variants differentially impact regulatory T cells and immune homeostasis. Immunology (2016) 148(3):276–86. doi: 10.1111/imm.12609 PMC4913290 27012310

[51] Safa K Chandran S Wojciechowski D. Pharmacologic targeting of regulatory T cells for solid organ transplantation: current and future prospects. Drugs (2015) 75(16):1843–52. doi: 10.1007/s40265-015-0487-6 26493288

[52] Scheinecker C Göschl L Bonelli M. Treg cells in health and autoimmune diseases: New insights from single cell analysis. J Autoimmun (2020) 110:102376. doi: 10.1016/j.jaut.2019.102376 31862128

