# Corrigendum: Mettl14-mediated m6A modification enhances the function of Foxp3^+^ regulatory T cells and promotes allograft acceptance

**DOI:** 10.3389/fimmu.2022.1112027

**Published:** 2022-12-20

**Authors:** Yanzhuo Liu, Yinglin Yuan, Zili Zhou, Yuanyuan Cui, Yan Teng, Hao Huang, Hao Yuan, Yanling Zhang, Lu Yang, Gaoping Zhao

**Affiliations:** ^1^ Department of Gastrointestinal Surgery, Sichuan Academy of Medical Sciences & Sichuan Provincial People’s Hospital, School of Medicine, University of Electronic Science and Technology of China, Chengdu, China; ^2^ Clinical Immunology Translational Medicine Key Laboratory of Sichuan Province, Sichuan Provincial People’s Hospital, University of Electronic Science and Technology of China, Chengdu, China; ^3^ Institute of Neurology, Sichuan Provincial People’s Hospital, School of Medicine, University of Electronic Science and Technology of China, Chengdu, China

**Keywords:** N6-methyladenosine, Mettl14, Treg function, transplantation, allograft acceptance

In the published article, there was an error in **Figure 4B** as published. In the original publication of this article, the same GraphPad file was accidentally linked in **Figures 4B, D**, which is a mistake. The corrected **Figure 4B** and its caption appear below.

**Figure 4:**
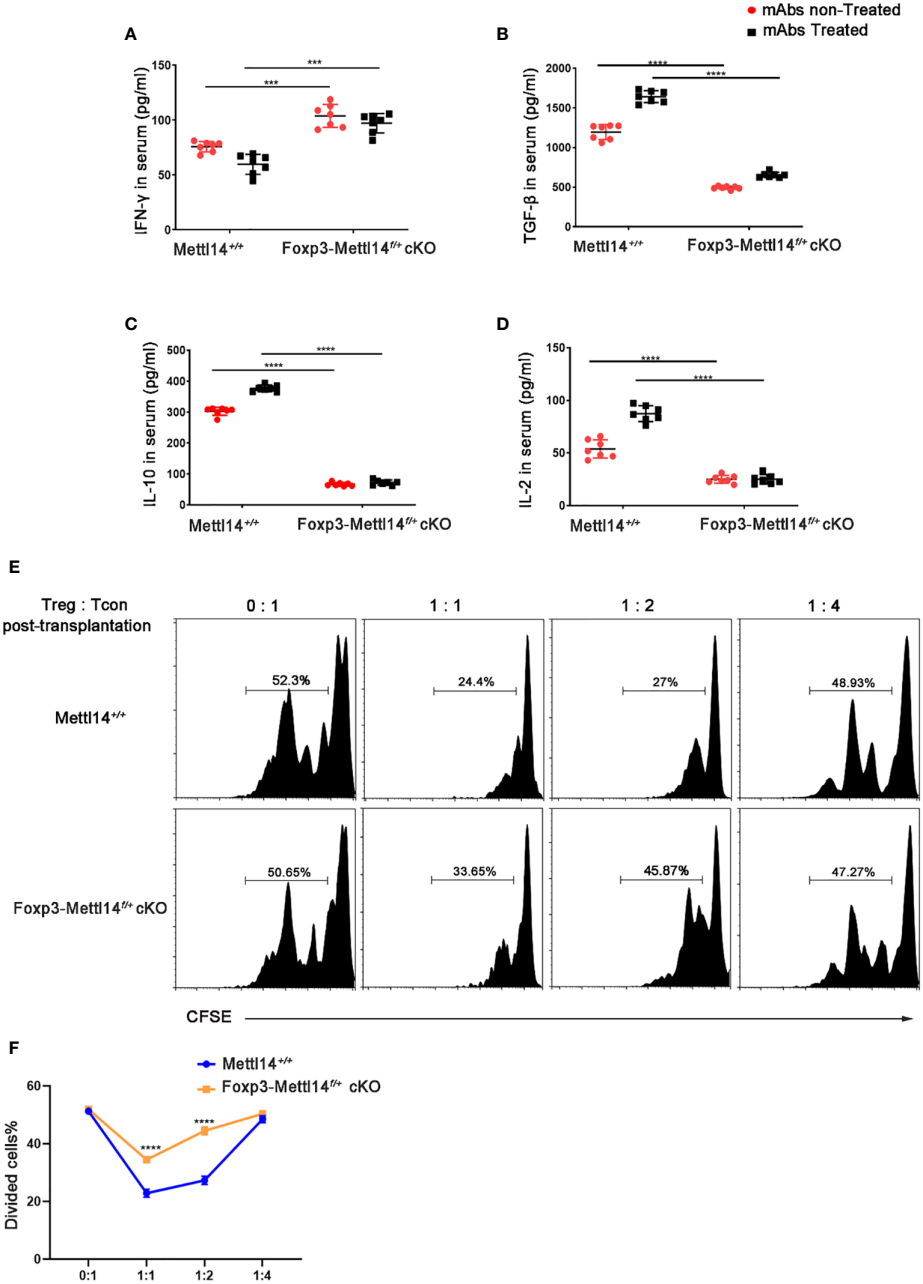
METTL14 deficiency Treg cells resulted in the loss of suppressive function after transplantation. **(A–D)** Serum was collected on day 7 after transplantation and cytokine levels were measured using ELISA assay. In the presence or absence of mAbs treatment, the scatter plots show the mean serum cytokine concentration in picograms per milliliter of IFN-γ **(A)**, TGF-β **(B)**, IL-10 **(C)** and IL-2 **(D)** from serum in Foxp3-Mettl14f/+ cKO mice and littermate controls (n=7). **(E)** The islets were isolated from BALB/c mice and transplanted them under the kidney capsule of littermate controls or Foxp3-Mettl14f/+ cKO mice with STZ-induced diabetes. These mice were treated with mAbs as described above. Treg cells were isolated on day 7 post-transplantation which were co-cultured with CFSE-labeled CD4+ naïve T cells from C57BL/6J mice, in round-bottom 96-well plates containing anti-CD3 (3 μg/ml) and anti-CD28 (5 μg/ml) monoclonal antibodies, at various ratios for 5 days. The suppressive effect on T cell expansion was detected using flow cytometry. **(F)** The suppressive function of Treg cells was quantified. The data are presented as the means ± SD. The data in **(A-D)** depict the mean values measured from seven separate experiments, while the data in **(D)** are representative of at least three independent experiments. The statistical analysis was performed with an unpaired Student’s t-test (two-tailed). ****P<0.0001 and ***P<0.001.

The authors apologize for this error and state that this does not change the scientific conclusions of the article in any way. The original article has been updated.

