# Supplementary material for: Mettl14-mediated m6A modification enhances the function of Foxp3+ regulatory T cells and promotes allograft acceptance
Source: Front Immunol. 2022 Oct 19;13:1022015. doi: 10.3389/fimmu.2022.1022015 (PMC9629694; doi:10.3389/fimmu.2022.1022015)
Supplement: Supplementary file 1 [file DataSheet_1.docx]

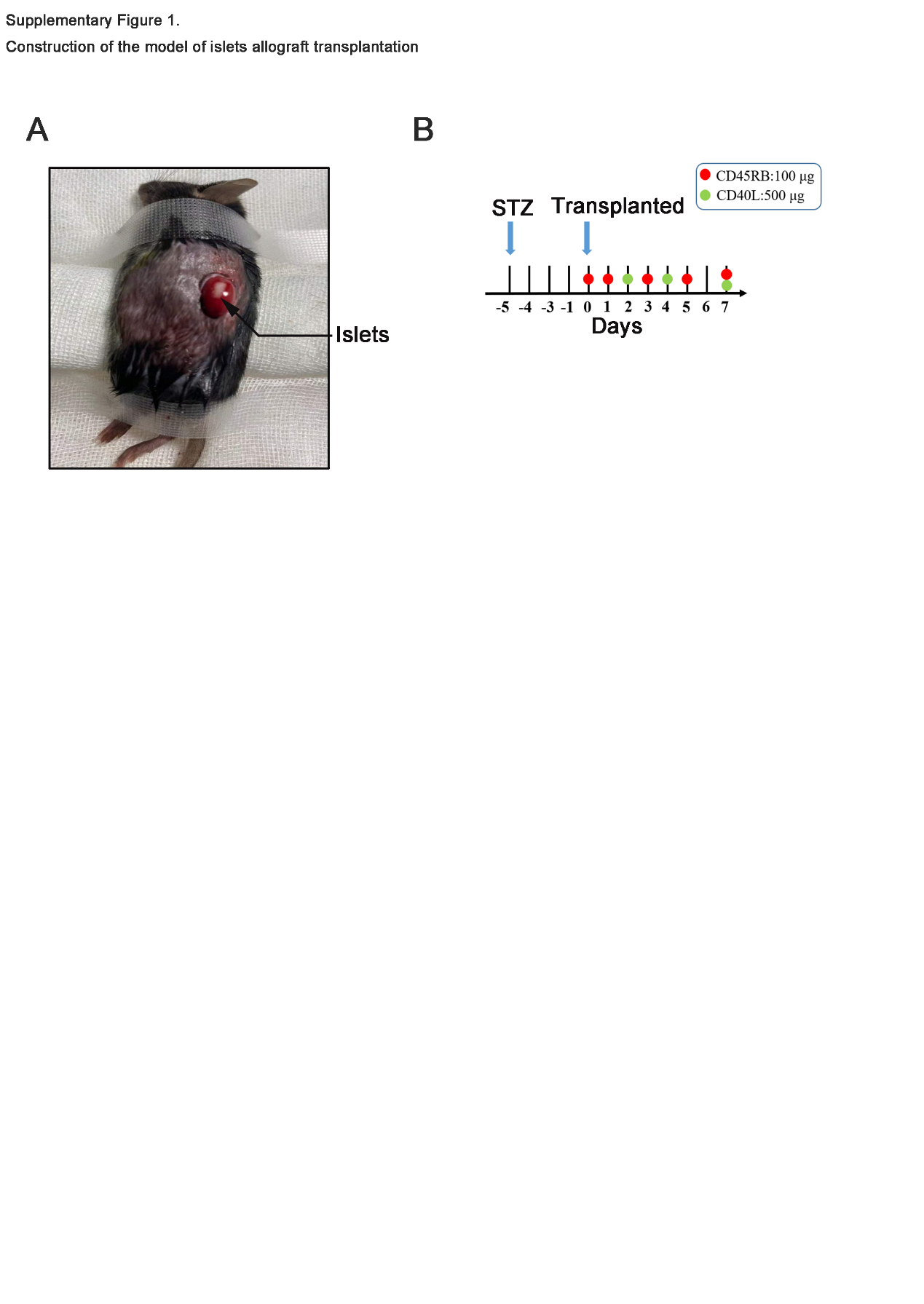


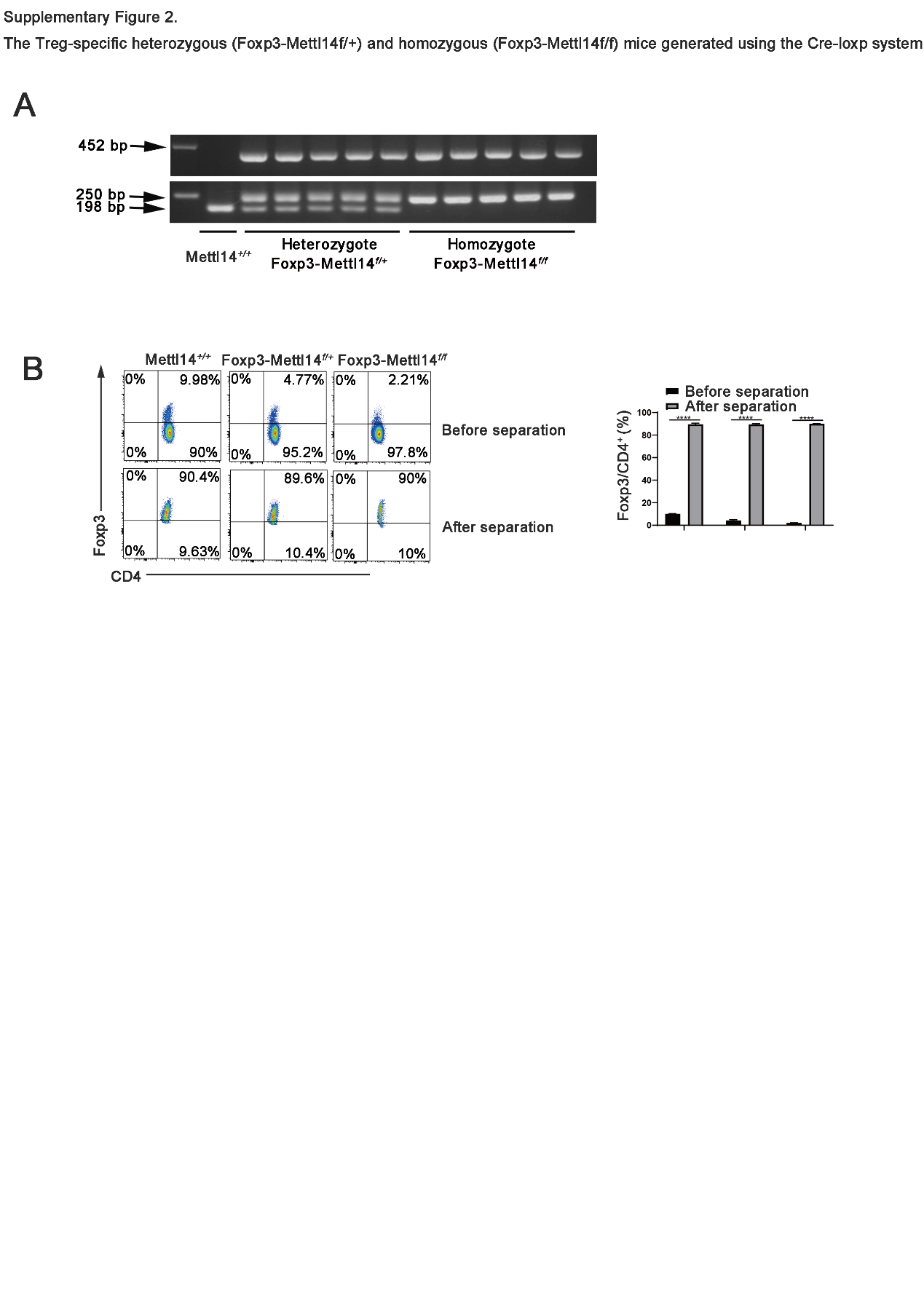


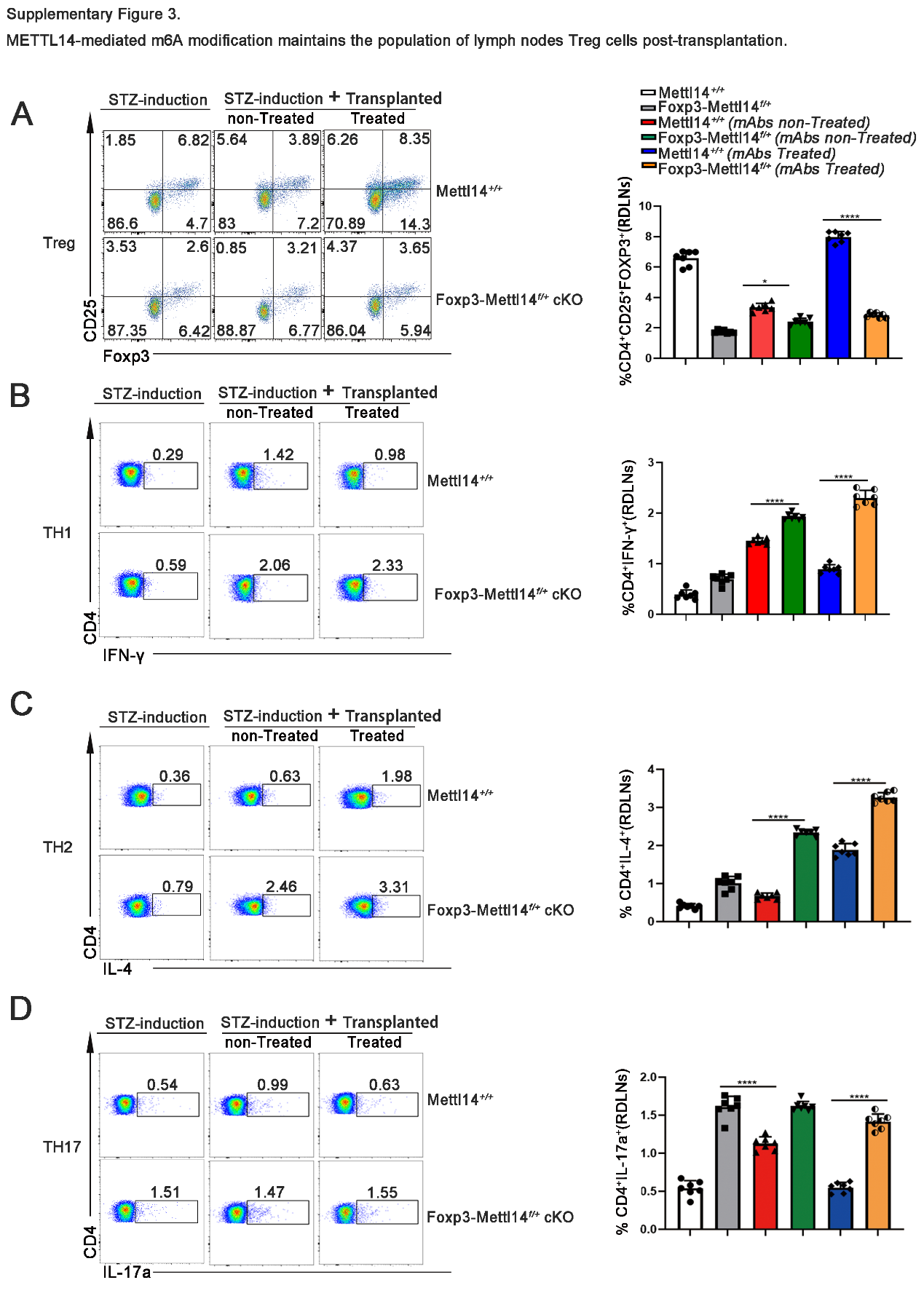


**Supplementary Figure 1**

**Construction of the model of islets allograft transplantation.**

(A) The model of allogeneic islet transplantation was constructed. (B) The plan of inducing the allograft immune tolerance by the anti-CD45RB and anti-CD40L monoclonal antibodies.

**Supplementary Figure 2**

**The Treg-specific heterozygous (Foxp3-Mettl14*^f/+^*) and homozygous (Foxp3-Mettl14*^f/f^*) mice generated using the Cre-loxp system**

(A) The homozygous and heterozygous mice were identified by genotyping results. (B) The purity of Treg was measured using flow cytometry (n = 10/group/experiment). The statistical analysis was performed with an unpaired Student’s T-test (two-tailed). ****P<0.0001.

**Supplementary Figure 3**

**METTL14-mediated m6A modification maintains the population of lymph nodes Treg cells post-transplantation.**

(A) In the presence or absence of mAbs, the proportion of CD4^+^CD25^+^Foxp3^+^ Treg cells from renal draining lymph nodes (RDLNs) was decreased in Foxp3-Mettl14*^f/+^* cKO mice compared to littermate controls after islets allograft transplantation. (B-D) Compared to littermate controls, the population of main subtypes of T-cell from renal draining lymph nodes including CD4^+^IFN-γ^+^TH1(B), CD4^+^IL-4^+^TH2(C) and CD4^+^IL-17a^+^TH17 (D) largely increased in Foxp3-Mettl14^f/+^ cKO mice after islets allograft transplantation. The data are shown as the means ± SD, the statistical analysis was performed with an unpaired Student’s t-test (two-tailed), ****P<0.0001, ***P<0.001 and ** P<0.01.
