# Supplementary material for: Mettl14-mediated m6A modification enhances the function of Foxp3+ regulatory T cells and promotes allograft acceptance
Source: Front Immunol. 2022 Oct 19;13:1022015. doi: 10.3389/fimmu.2022.1022015 (PMC9629694; doi:10.3389/fimmu.2022.1022015)
Supplement: Supplementary file 2 [file Table_1.docx]

| ID | Type | ID | Type |
| --- | --- | --- | --- |
| Shank1 | UP | Shank3 | DOWN |
| Syt6 | UP | Syt7 | DOWN |
| Dag1 | UP | Car2 | DOWN |
| Cebpb | UP | Rgs11 | DOWN |
| Trem1 | UP | Slc30a10 | DOWN |
| Cma1 | UP | Tac2 | DOWN |
| Ralgps2 | UP | Ribc1 | DOWN |
| Cd302 | UP | Hacd1 | DOWN |
| Pold4 | UP | Auts2 | DOWN |
| Rnaset2a | UP | Abcb10 | DOWN |
| Nutm2 | UP | Muc13 | DOWN |
| Tmem163 | UP | Peg12 | DOWN |
| Itgad | UP | Igsf3 | DOWN |
| Cd36 | UP | Tnfsfm13 | DOWN |
| Itga9 | UP | Gm5134 | DOWN |
| Acsf2 | UP | Epdr1 | DOWN |
| Zfp867 | UP | Ifrd2 | DOWN |
| Zfp12 | UP | Hbb-b1 | DOWN |
| Ebf1 | UP | Pklr | DOWN |
| Shroom2 | UP | Mrpl47 | DOWN |
| Sirpb1c | UP | Smim1 | DOWN |
| Cxcr5 | UP | Tfr2 | DOWN |
| Rgs13 | UP | Asb16 | DOWN |
| Pkib | UP | Zfp275 | DOWN |
| Ccdc50 | UP | Myh10 | DOWN |
| Hbb-bs | UP | Lmod1 | DOWN |
| Cpt1a | UP | Ces1d | DOWN |
| Ptprm | UP | Gja1 | DOWN |
| H2-Eb1 | UP | Ahrr | DOWN |
| H2-Eb2 | UP | Dnajc6 | DOWN |
| Map3k8 | UP | Fam124a | DOWN |
| Map3k9 | UP | Foxe1 | DOWN |
| Abca1 | UP | Tbc1d19 | DOWN |
| Ltk | UP | Clcn2 | DOWN |
| Abca9 | UP | Vldlr | DOWN |
| Etv3 | UP | Adgrg7 | DOWN |
| Sh3bp2 | UP | Cdc6 | DOWN |
| Rgs2 | UP | Aqp1 | DOWN |
| Tcn2 | UP | Hephl1 | DOWN |
| Olfr804 | UP | Plcxd1 | DOWN |
| Zfp36 | UP | Myl12a | DOWN |
| Pik3c2b | UP | Car1 | DOWN |
| Dock4 | UP | Abcg4 | DOWN |
| Tlr13 | UP | Thop1 | DOWN |
| Zfp318 | UP | Tjp1 | DOWN |
| Rnf122 | UP | Prdx2 | DOWN |
| Gm20498 | UP | 1110002E22Rik | DOWN |
| Tspan14 | UP | Mt1 | DOWN |
| Pfkfb1 | UP | Gosr1 | DOWN |
| Fstl3 | UP | C530008M17Rik | DOWN |
| Plk2 | UP | Mgst3 | DOWN |
| Prag1 | UP | Arf3 | DOWN |
| Lrrc32 | UP | Lhx1 | DOWN |
| Abcc3 | UP | Stra6 | DOWN |
| Gm8369 | UP | Evc2 | DOWN |
| Slc25a20 | UP | Grb10 | DOWN |
| Rnase6 | UP | Ank1 | DOWN |
| Hvcn1 | UP | Pbk | DOWN |
| Aicda | UP | Slc25a44 | DOWN |
| Rnf146 | UP | Pheta2 | DOWN |
| Mlst8 | UP | Tspan8 | DOWN |
| Stab2 | UP | Pm20d2 | DOWN |
| H2-Q10 | UP | Ache | DOWN |
| Pik3ap1 | UP | Ercc6l | DOWN |
| Galns | UP | Chtop | DOWN |
| Evi2 | UP | Gstm5 | DOWN |
| Fgfr1op | UP | Fignl1 | DOWN |
| C1qb | UP | Cpox | DOWN |
| Smagp | UP | Dnase2a | DOWN |
| Slc11a1 | UP | St6galnac6 | DOWN |
| H2-Ab1 | UP | Espnl | DOWN |
| Fam129c | UP | Fut10 | DOWN |
| Zscan26 | UP | Sparcl1 | DOWN |
| Mef2c | UP | Cd34 | DOWN |
| H2-DMb1 | UP | Shisa9 | DOWN |
| Fcmr | UP | Sept8 | DOWN |
| Ikzf3 | UP | Cyth3 | DOWN |
| Apobec1 | UP | Sgcg | DOWN |
| Hip1 | UP | Gm21992 | DOWN |
| Kctd12b | UP | Tpm2 | DOWN |
| Pira2 | UP | Tom1l1 | DOWN |
| Egr3 | UP | Eri2 | DOWN |
| Fcna | UP | Lpo | DOWN |
| Gnal | UP | Paqr9 | DOWN |
| Osm | UP | Dmtn | DOWN |
| Tgoln2 | UP | Gypa | DOWN |
| Stfa2l1 | UP | Psma8 | DOWN |
| Rubcn | UP | Slc25a21 | DOWN |
| Usp44 | UP | Prtn3 | DOWN |
| Blk | UP | Mecom | DOWN |
| Galr2 | UP | Sh3tc2 | DOWN |
| Tnfsf13 | UP | Trim6 | DOWN |
| Tnfsf12 | UP | Vwa1 | DOWN |
| Slc41a2 | UP | Adk | DOWN |
| B3gnt5 | UP | Adgrg1 | DOWN |
| Adap2 | UP | Grm1 | DOWN |
| Gpr35 | UP | Maoa | DOWN |
| Hpgd | UP | Chrnb4 | DOWN |
| Tmem51 | UP | Arhgap29 | DOWN |
| Moap1 | UP | Grk1 | DOWN |
| Arf1 | UP | Atp7b | DOWN |
| Siglecg | UP | Add2 | DOWN |
| Zbtb25 | UP | Klhl26 | DOWN |
| Sbk1 | UP | Klhl23 | DOWN |
| 5430427O19Rik | UP | Ackr1 | DOWN |
| Ddn | UP | Fads3 | DOWN |
| Adgrg5 | UP | Fads2 | DOWN |
| Cd74 | UP | Adgra3 | DOWN |
| Arrdc4 | UP | Tmem170b | DOWN |
| Ell3 | UP | Aldh1l1 | DOWN |
| Hcar2 | UP | Sox6 | DOWN |
| Trim7 | UP | Adamts5 | DOWN |
| Clec4a1 | UP | Mt2 | DOWN |
| Ptprj | UP | Pkhd1l1 | DOWN |
| Hs3st1 | UP | Eps8l3 | DOWN |
| Junb | UP | Rpf2 | DOWN |
| Scpep1 | UP | Orc2 | DOWN |
| Plcl1 | UP | Micall2 | DOWN |
| Nova1 | UP | Spata31d1b | DOWN |
| Cacna1i | UP | Ccl28 | DOWN |
| Gpsm1 | UP | Osbpl1a | DOWN |
| Serpinb10 | UP | Col5a1 | DOWN |
| Cd5l | UP | Cttn | DOWN |
| Shroom3 | UP | Jag2 | DOWN |
| Rhbdf1 | UP | Bnc2 | DOWN |
| Scimp | UP | Rab3d | DOWN |
| Marcks | UP | Car13 | DOWN |
| Slc16a9 | UP | Pycr1 | DOWN |
| Siglece | UP | Spire1 | DOWN |
| Sirpb1b | UP | Spire2 | DOWN |
| Zfp608 | UP | Hnf4a | DOWN |
| Slc35a5 | UP | Nags | DOWN |
| Zfp182 | UP | Chchd6 | DOWN |
| Xkrx | UP | Fabp5 | DOWN |
| F11r | UP | 4921536K21Rik | DOWN |
| H2-DMb2 | UP | Sdsl | DOWN |
| Map3k19 | UP | Plek2 | DOWN |
| Erp27 | UP | Acsl6 | DOWN |
| Rsph4a | UP | Tfrc | DOWN |
| Arhgap26 | UP | Rhd | DOWN |
| Pira1 | UP | Zfp708 | DOWN |
| Ifi204 | UP | Icam5 | DOWN |
| Cd300a | UP | Ctsg | DOWN |
| Cabp7 | UP | Cdca2 | DOWN |
| Susd3 | UP | Caskin2 | DOWN |
| Cdh17 | UP | Syde2 | DOWN |
| Adgre1 | UP | Trim67 | DOWN |
| Ly86 | UP | Espn | DOWN |
| Vcam1 | UP | C030006K11Rik | DOWN |
| 1700001O22Rik | UP | Hsph1 | DOWN |
| E2f2 | UP | Kel | DOWN |
| Ccr3 | UP | Fhit | DOWN |
| Tmem26 | UP | Dixdc1 | DOWN |
| Tuba4a | UP | Gm14296 | DOWN |
| Pex11g | UP | Abcb4 | DOWN |
| Slc25a25 | UP | Phldb1 | DOWN |
| Axl | UP | Slc6a20a | DOWN |
| Cmklr1 | UP | Gatm | DOWN |
| Dnah7a | UP | Cdc25c | DOWN |
| Zc3h12c | UP | Epor | DOWN |
| Tpd52 | UP | Esrrg | DOWN |
| Gm9733 | UP | Gpr1 | DOWN |
| Mertk | UP | Hs6st2 | DOWN |
| Gxylt1 | UP | Cldn13 | DOWN |
| Cr2 | UP | Cplane2 | DOWN |
| Cd72 | UP | Tmem97 | DOWN |
| Rabep2 | UP | Ces2g | DOWN |
| Nfkbiz | UP | Tspan33 | DOWN |
| SOCS1 | UP | Slc43a1 | DOWN |
| SOCS3 | UP | Exo1 | DOWN |
| SOCS2 | UP | Gfi1b | DOWN |
| Adrb2 | UP | Adgrl4 | DOWN |
| Edaradd | UP | Adgrl2 | DOWN |
| Atf3 | UP | Sspo | DOWN |
| Clu | UP | Spin4 | DOWN |
| Sema4d | UP | Disp2 | DOWN |
| Klhl24 | UP | Wipf3 | DOWN |
| Rag1 | UP | Pira7 | DOWN |
| Lat2 | UP | Pira4 | DOWN |
| Cd79b | UP | C1qtnf12 | DOWN |
| Cd79a | UP | Ildr2 | DOWN |
| Ceacam16 | UP | Pclo | DOWN |
| Whrn | UP | Ubac1 | DOWN |
| Timd4 | UP | LOC114841036 | DOWN |
| H2-Oa | UP | Scml2 | DOWN |
| Tlr8 | UP | Gata1 | DOWN |
| Tlr9 | UP | Tceal8 | DOWN |
| Sik1 | UP | Cep290 | DOWN |
| Hmox1 | UP | Zfp382 | DOWN |
| Hhex | UP | Adamts20 | DOWN |
| S1pr3 | UP | Amd1 | DOWN |
| Tbxa2r | UP | Slc38a5 | DOWN |
| Sycp2 | UP | Psmd9 | DOWN |
| Ctsh | UP | Ptdss2 | DOWN |
| Cds2 | UP | Sec14l2 | DOWN |
| Cd163l1 | UP | Klhl4 | DOWN |
| Calhm5 | UP | Pla2g4f | DOWN |
| Fcrl1 | UP | Olfr419 | DOWN |
| Fcrl5 | UP | Nwd1 | DOWN |
| Rasgef1b | UP | Fn3krp | DOWN |
| Fcrla | UP | Tpbg | DOWN |
| Trpm2 | UP | Fads1 | DOWN |
| Lgmn | UP | Smo | DOWN |
| Swap70 | UP | Sptb | DOWN |
| Nfam1 | UP | Adgrf5 | DOWN |
| Rab36 | UP | Hmga1 | DOWN |
| Rab30 | UP | Atp1b2 | DOWN |
| Tnfaip8 | UP | Epb42 | DOWN |
| Cyp27a1 | UP | Syce2 | DOWN |
| Pdgfc | UP | Trip13 | DOWN |
| Pira11 | UP | Pde1c | DOWN |
| Ms4a4a | UP | Clec2g | DOWN |
| Trnp1 | UP | Igfn1 | DOWN |
| Ly6d | UP | Zfp322a | DOWN |
| Trim30d | UP | Cited4 | DOWN |
| Anks6 | UP | Pcdh12 | DOWN |
| BC028528 | UP | Plscr4 | DOWN |
| Tlr7 | UP | Mtfp1 | DOWN |
| Htd2 | UP | Slc6a9 | DOWN |
| Itgb5 | UP | Mns1 | DOWN |
| Dusp1 | UP | Gpc4 | DOWN |
| Arhgef37 | UP | Bbs7 | DOWN |
| Fcgr1 | UP | Prkar2b | DOWN |
| Ston1 | UP | Kctd16 | DOWN |
| Pxdc1 | UP | Layn | DOWN |
| Carmil1 | UP | Tmem132b | DOWN |
| Bcar3 | UP | Evc | DOWN |
| Sorl1 | UP | Zfp982 | DOWN |
| Ctrb1 | UP | Rhag | DOWN |
| Igf1 | UP | Tmem33 | DOWN |
| Hhat | UP | Pgr | DOWN |
| Pla2g7 | UP | Ttc41 | DOWN |
| Cxcl2 | UP | Cd248 | DOWN |
| Klf15 | UP | Hhip | DOWN |
| Klhl14 | UP | Slc4a1 | DOWN |
| Carns1 | UP | Igf2bp1 | DOWN |
| Bbc3 | UP | Efcc1 | DOWN |
| Cerk | UP | Klhdc7b | DOWN |
| Gm1966 | UP | Stxbp1 | DOWN |
| Klra17 | UP | Tal1 | DOWN |
| Msr1 | UP | Vma21 | DOWN |
| Setbp1 | UP | Hist1h2ao | DOWN |
| Lurap1 | UP | 1110065P20Rik | DOWN |
| C6 | UP | Klf1 | DOWN |
| Myof | UP | Ninl | DOWN |
| Gns | UP | Nipa1 | DOWN |
| Bfsp2 | UP | Hemgn | DOWN |
| Plk3 | UP | Chid1 | DOWN |
| Tmem240 | UP | Cd200r3 | DOWN |
| Ccr1 | UP | Has1 | DOWN |
| Ccr6 | UP | Abca17 | DOWN |
| Vcan | UP | Hebp1 | DOWN |
| Slc25a53 | UP | Btnl10 | DOWN |
| Il12a | UP | Mpo | DOWN |
| Csf1r | UP | Fam83c | DOWN |
| Hlx | UP | Fam83d | DOWN |
| Clec4n | UP | 1300017J02Rik | DOWN |
| Cd55 | UP | Cenpm | DOWN |
| Cd8b1 | UP | Cenpk | DOWN |
| Klhl6 | UP | Epx | DOWN |
| Ptgs2 | UP | Asns | DOWN |
| Pdzd4 | UP | Samd11 | DOWN |
| Pax5 | UP | Cdr2 | DOWN |
| Slc9a7 | UP | Rec8 | DOWN |
| Slc29a4 | UP | Tpmt | DOWN |
| Fos | UP | Steap3 | DOWN |
| Srpk3 | UP | Mfsd2a | DOWN |
| Slco2b1 | UP | Kcna6 | DOWN |
| Lrp1 | UP | Ephb2 | DOWN |
| Sorcs1 | UP | Kbtbd12 | DOWN |
| Ttyh2 | UP | Acyp1 | DOWN |
| Neb | UP | Hmbs | DOWN |
| Fcer2a | UP | Aldh1a1 | DOWN |
| Egr1 | UP | Aldh1a2 | DOWN |
| Tnfrsf13c | UP | Ermap | DOWN |
| Tspan17 | UP | Bmp6 | DOWN |
| Treml4 | UP | Chst1 | DOWN |
| Gcnt2 | UP |  |  |
| Zcchc24 | UP |  |  |
| Apoe | UP |  |  |
| Acod1 | UP |  |  |
| Stac2 | UP |  |  |
| Snai1 | UP |  |  |
| Ciita | UP |  |  |
| Fn1 | UP |  |  |
| Haao | UP |  |  |
| Sfxn5 | UP |  |  |
| Blnk | UP |  |  |
| Fam43a | UP |  |  |
| Slc6a19 | UP |  |  |
| Slc6a12 | UP |  |  |
| Btla | UP |  |  |
| Slc43a2 | UP |  |  |
| Ccl6 | UP |  |  |
| Mdm4 | UP |  |  |
| Nlrp3 | UP |  |  |
| Mpeg1 | UP |  |  |
| Mrgpre | UP |  |  |
| Entpd3 | UP |  |  |
| Phka1 | UP |  |  |
| Cd40 | UP |  |  |
| Icosl | UP |  |  |
| Snn | UP |  |  |
| H2-Aa | UP |  |  |
| Csrnp1 | UP |  |  |
| Themis2 | UP |  |  |
| Nr4a1 | UP |  |  |
| Wipf1 | UP |  |  |
| Il1r2 | UP |  |  |
| Nuak2 | UP |  |  |
| Eif3j1 | UP |  |  |
| F5 | UP |  |  |
| Psd3 | UP |  |  |
| Unc93b1 | UP |  |  |
| Thbs1 | UP |  |  |
| Klra18 | UP |  |  |
| Rubcnl | UP |  |  |
| Cd180 | UP |  |  |
| Cers6 | UP |  |  |
| Tubb1 | UP |  |  |
| Tmed6 | UP |  |  |
| Cstdc5 | UP |  |  |
| Bmf | UP |  |  |
| Clec7a | UP |  |  |
| Tspan15 | UP |  |  |
| Vpreb3 | UP |  |  |
| Adora3 | UP |  |  |
| Tnfaip3 | UP |  |  |
| Pou2f2 | UP |  |  |
| Mafb | UP |  |  |
| Kmo | UP |  |  |
| Mrc1 | UP |  |  |
| Tcf7l2 | UP |  |  |
| Ace | UP |  |  |
| Evi2b | UP |  |  |
| Ier5 | UP |  |  |
| Myo1e | UP |  |  |
| Spib | UP |  |  |
| Spic | UP |  |  |
| March1 | UP |  |  |
| Lyn | UP |  |  |
| H2ac19 | UP |  |  |
| Irf5 | UP |  |  |
| Zbtb10 | UP |  |  |
| Zbtb18 | UP |  |  |
| Mlxip | UP |  |  |
| Il5ra | UP |  |  |
| Tbxas1 | UP |  |  |
| Ganc | UP |  |  |
| Syk | UP |  |  |
| Lynx1 | UP |  |  |
| Sdc3 | UP |  |  |
| Sdc4 | UP |  |  |
| Camk1d | UP |  |  |
| Pltp | UP |  |  |
| Sat1 | UP |  |  |
| Lifr | UP |  |  |
| Tctn1 | UP |  |  |
| Fosb | UP |  |  |
| H2-DMa | UP |  |  |
| Nr1h3 | UP |  |  |
| Zmym1 | UP |  |  |
| Khdc1c | UP |  |  |
| Cdkl1 | UP |  |  |
| Rnf170 | UP |  |  |
| 4921507P07Rik | UP |  |  |
| Map1lc3a | UP |  |  |
| Cyp2ab1 | UP |  |  |
| Dnmt3a | UP |  |  |
| C1qc | UP |  |  |
| Scd1 | UP |  |  |
| Cd19 | UP |  |  |
| Osgin1 | UP |  |  |
| Pgap1 | UP |  |  |
| Klra8 | UP |  |  |
| Klra2 | UP |  |  |
| Zfp672 | UP |  |  |
| Lrrn4cl | UP |  |  |
| Snx29 | UP |  |  |
| Plek | UP |  |  |
| C4b | UP |  |  |
| Smpdl3b | UP |  |  |
| Fgd4 | UP |  |  |
| Lrrk2 | UP |  |  |
| Bank1 | UP |  |  |
| Plxnb2 | UP |  |  |
| Il1b | UP |  |  |
| Smim14 | UP |  |  |
| Adamdec1 | UP |  |  |
| Dennd3 | UP |  |  |
| C1qa | UP |  |  |
| Smoc1 | UP |  |  |
| Jun | UP |  |  |
| Jup | UP |  |  |
| Ifi207 | UP |  |  |
| Ldlrad3 | UP |  |  |
| Prkcb | UP |  |  |
| Cx3cr1 | UP |  |  |
| Tsc22d3 | UP |  |  |
| Cd22 | UP |  |  |
| Gfra2 | UP |  |  |
| Zfp90 | UP |  |  |
| Casz1 | UP |  |  |
| Prr5 | UP |  |  |
| Sash1 | UP |  |  |
| Il7 | UP |  |  |
| Dlx2 | UP |  |  |
| Coq8a | UP |  |  |
| Ms4a1 | UP |  |  |
| C5ar1 | UP |  |  |
| Iqsec1 | UP |  |  |
| Emid1 | UP |  |  |
| Fcgr4 | UP |  |  |
| Tmod1 | UP |  |  |
| Pmaip1 | UP |  |  |
| Dok3 | UP |  |  |
| Snx30 | UP |  |  |
| Cyp4f18 | UP |  |  |
| Jade2 | UP |  |  |
| Cd38 | UP |  |  |
| Tsga13 | UP |  |  |
| Plaur | UP |  |  |
| Il9r | UP |  |  |
| Htr1d | UP |  |  |
| Gm6377 | UP |  |  |
| Misp3 | UP |  |  |
| Cd8a | UP |  |  |
| Cd80 | UP |  |  |
| Cd86 | UP |  |  |
| Cyfip2 | UP |  |  |
| Zc3h6 | UP |  |  |
| Adgre4 | UP |  |  |
| Fhl1 | UP |  |  |
| Chst3 | UP |  |  |
